# Vision-Based Structural Health Monitoring of Catenary Components in UAV Inspections via Mask-Guided Asymmetrical Flow

**DOI:** 10.3390/biomimetics11090679

**Published:** 2026-09-20

**Authors:** Qiaolu Wang, Haitao Lan, Tianyu Zhou, Ning Ma, Haonan Yang, Jingke Yan

**Affiliations:** 1 School of Artificial Intelligence and Electronic Engineering, Sichuan Technology and Business University, No. 65, Xueyuan Road, Pidu District, Chengdu 611745, China; 212017083500005@stu.xhu.edu.cn; 2 School of Electrical Engineering, Southwest Jiaotong University, Chengdu 610097, China; zhoutianyu@my.swjtu.edu.cn (T.Z.); mn328898782@my.swjtu.edu.cn (N.M.); hnyang@my.swjtu.edu.cn (H.Y.); 3 State Key Laboratory of Rail Transit Vehicle System, Southwest Jiaotong University, Chengdu 610000, China; yanjingke@my.swjtu.edu.cn

**Keywords:** structural health monitoring (SHM), UAV inspection, catenary components, unsupervised learning, environmental and operational variations (EOVs), normalizing flow, damage localization

## Abstract

Automated vision-based structural health monitoring (SHM) of catenary support components is critical for ensuring railway operational safety. However, achieving reliable structural damage identification in practical engineering scenarios is hindered by complex environmental and operational variations (EOVs) in aerial imagery, the multi-scale nature of structural degradation, and the scarcity of damage samples. To address these SHM challenges, this paper proposes a high-precision unsupervised damage detection framework named Mask-Guided Asymmetrical Flow (MGAF). First, to mitigate the impact of EOVs, a SAM-guided preprocessing strategy is introduced to explicitly suppress background interference and extract effective structural Regions of Interest (ROIs). Second, an Asymmetrical Parallel Flow architecture is designed to balance damage detection sensitivity and processing latency. By optimizing the flow depth for high-resolution features, this architecture prevents overfitting to high-frequency environmental noise while preserving deep semantic information. Furthermore, a Cross-Scale Feature Fusion (CSFF) module is developed to enhance the detection capability for early-stage structural damages (e.g., fatigue micro-cracks and fastener looseness) by integrating global structural semantics with local textural damage indicators. Finally, a Morphological Edge Suppression (MES) mechanism is employed to eliminate boundary artifacts, thereby reducing the false alarm rate in pixel-level damage localization. Extensive experiments on the self-constructed CSCUD dataset demonstrate that the proposed system achieves competitive anomaly detection performance compared with representative unsupervised methods with an Image-level AUROC of 98.9% and a Pixel-level AP of 40.1%. During online inference, MGAF achieves a processing time of 0.077 s per frame on an NVIDIA RTX 4090 GPU, excluding the offline GSI-Net localization and SAM-based structural ROI extraction stages. This demonstrates the efficiency of the proposed anomaly detection module for practical railway inspection scenarios. Additional experiments on selected categories from the MVTec AD benchmark provide preliminary evidence of the transferability of MGAF to visually similar industrial anomaly detection scenarios.

## 1. Introduction

The support components of railway catenary systems act as the primary load-bearing structures for the overhead infrastructure, playing a pivotal role in maintaining the mechanical stability and precise spatial geometry required for train operations. During service, these components are continuously subjected to intense aerodynamic forces, high-frequency vibrations, and severe dynamic impacts generated by the high-speed movement of trains. Prolonged exposure to such harsh operational loads and complex environmental weathering inevitably accelerates structural degradation, leading to fatigue-induced micro-cracks, fastener looseness, or even complete mechanical failure [1,2]. Consequently, the early detection and precise quantification of these structural damages represent a critical structural health monitoring (SHM) task to prevent catastrophic infrastructural collapse and ensure public safety.

Traditional condition assessment methods primarily rely on manual visual patrols, which are not only inefficient and subjective, but also inadequate for coping with the challenges of complex outdoor environments. The deployment of next-generation vision-based non-contact monitoring systems—such as inspection vehicles and unmanned aerial vehicles (UAVs) equipped with robust visual–inertial navigation and real-time spatial tracking capabilities [3]—has significantly reduced operation and maintenance costs. However, due to limitations in camera angles and shooting distances, coupled with the extreme scarcity of actual damage samples and the diverse manifestations of structural degradation, existing automated fault diagnosis algorithms still struggle to meet the stringent requirements for high precision and early warning capabilities in practical condition-based maintenance (CBM) applications.

Existing damage detection methods for catenary components can be broadly categorized into traditional computer vision algorithms and deep learning-based approaches. Traditional algorithms typically follow a “feature extraction–template matching” pipeline to identify structural faults. For instance, Zhang et al. [4] employed Harris corner detection to extract image features and utilized sequence similarity algorithms to locate targets; Han et al. [5] used affine-invariant moment matching to localize insulators and performed multi-scale analysis for defect identification; Karabase et al. [6] proposed a fault diagnosis method for the pantograph–catenary system based on fuzzy logic. While these approaches are computationally efficient, their feature extraction processes rely heavily on handcrafted designs, rendering them highly susceptible to environmental and operational variations (EOVs), thereby severely limiting their generalization capabilities in field environments.

In recent years, deep learning-based methods have become dominant in the condition assessment of catenary components, often adopting a “localization-then-damage-detection” paradigm. For example, Zhong et al. [7] introduced the TOL framework, which combines precise localization with adversarial reconstruction to assess component conditions; Zhang et al. [8] developed the ECF-STPM network, integrating a self-calibrated illumination enhancement module to detect structural surface cracks; Chen et al. [9] designed a cascaded neural network for fastener fault detection; and Xie et al. [10] proposed MTA-Net, which achieves multi-task alignment across scales. In recent years, extensive research has been conducted on the health monitoring of complex mechanical systems and infrastructure, with particular emphasis on feature enhancement, adaptation to complex operating conditions, and fine-grained damage identification [11]. Alongside traditional optical methods, non-contact sensing technologies and high-fidelity imaging—such as noise-optimized resonant sensors for metallic stress estimation [12] and advanced terahertz image super-resolution for preserving critical structural details [13,14]—have been vigorously developed to advance non-destructive testing (NDT). Some studies have focused on fault diagnosis of rotating machinery, employing hybrid attention mechanisms, wide-convolution denoising, and spatio-temporal feature mining strategies to improve the discriminative capability and generalization performance of vibration signals under small-sample conditions. Other works have integrated variational mode decomposition, deep feature fusion, and ensemble learning schemes to enhance diagnostic stability in nonlinear and non-stationary signal scenarios [15]. To address the issue of data distribution shift under complex operating conditions, some researchers have further introduced multiscale graph representation learning and prototype-assisted mechanisms to improve the robustness of unsupervised state recognition [16]. Meanwhile, in the field of vision-based health monitoring for infrastructure, de-shadowing modules, instance segmentation networks, and geometrical morphology analysis have been adopted to improve pixel-level damage identification accuracy and structural state quantification under complex environments [17]. Furthermore, methodological advancements in adjacent deep learning domains—such as multimodal image fusion via cross-attention [18] and adaptive 3D segmentation networks for complex image analysis [19]—have provided valuable architectural inspiration for extracting intricate structural features from noisy visual data. Overall, existing studies indicate that multiscale feature modeling, suppression of environmental interference, and the collaborative representation of local damage and global structural information are key directions for improving the engineering applicability of intelligent health monitoring systems [20]. However, despite their impressive performance, these supervised learning methods face a fundamental bottleneck in real-world SHM applications: the extreme scarcity of anomalous structural samples, which makes it exceedingly difficult to construct the balanced and representative training datasets required for reliable fault diagnosis.

To address the inherent scarcity of structural damage samples, Unsupervised Damage Detection (UDD) has emerged as a highly promising paradigm for structural health monitoring. Similar to the fundamental challenges faced in complex video surveillance for abnormal activity detection and robust multi-object tracking [21,22], isolating sparse anomalies from dynamic, cluttered backgrounds requires powerful generative or spatial-temporal feature representations. Early studies explored condition assessment through spatial alignment and contrastive learning strategies [23,24]. More recently, generative approaches based on Normalizing Flows have gained significant attention due to their ability to explicitly model the probability density of healthy structural states. For example, FastFlow and CS-Flow achieve high-performance localization using 2D flow-based models. To further support the monitoring of multiple structural components, You et al. [25] proposed UniAD, followed by unified frameworks such as MambaAD [26], which further reduce deployment complexity. Recently, Yang et al. [27] combined the Segment Anything Model (SAM) with a prototype-based network (INRP-ADer) and made notable progress in addressing feature inconsistencies caused by varying field inspection conditions.

Despite the promising results of these vision-based non-destructive evaluation (NDE) methods, applying flow-based generative models to aerial imagery of catenary systems captured by UAVs introduces three fundamental SHM challenges—challenges that this study seeks to address:

(1) Susceptibility to Environmental and Operational Variations (EOVs): Unlike controlled industrial datasets (e.g., MVTec AD), aerial images of catenary systems collected in the field contain complex environmental clutter such as gravel, rails, and vegetation. Standard flow-based models tend to overfit these EOVs, resulting in a high false alarm rate (FAR) during condition assessment. While foreground extraction techniques may alleviate this issue, efficiently and cleanly isolating the structural Region of Interest (ROI) from complex outdoor backgrounds remains a nontrivial task.
(2) Overfitting to high-frequency noise and degradation of damage indicators: Accurate quantification of early-stage structural degradation relies on high-resolution shallow features. However, existing multi-scale flow models often adopt symmetric architectures, applying uniform flow depths across all scales. This design proves suboptimal for field-acquired imagery, where high-resolution features inherently contain significant high-frequency noise—such as sensor artifacts and textural EOVs. Applying deep flow transformations to these features leads to overfitting, where random environmental noise is incorrectly modeled as part of the baseline “healthy” distribution. This not only impairs the model’s sensitivity to genuine structural anomalies but also significantly increases false alarms.
(3) Localization ambiguity for early-stage structural damages: During deep feature extraction, the spatial details of small-scale structural damages—such as fatigue micro-cracks or missing fasteners—are prone to degradation, resulting in blurred damage localization heatmaps. Consequently, the pixel-level average precision (P-AP) remains difficult to improve, posing a significant obstacle for the precise geometric segmentation and quantitative evaluation of early-stage structural faults.

Building upon the above analysis, this paper proposes a high-precision unsupervised damage detection framework named MGAF (Mask-Guided Asymmetrical Flow) for structural health monitoring. First, we introduce a SAM-guided preprocessing strategy, which leverages component location information to explicitly eliminate environmental and operational variations (EOVs), thereby extracting clean structural Regions of Interest (ROIs) for reliable condition assessment. Second, we design an asymmetrical parallel flow architecture that reduces the flow depth for high-resolution features. This design effectively prevents the model from overfitting to high-frequency environmental noise while significantly improving the processing speed to meet the requirements of real-time early warning. Finally, by integrating Cross-Scale Feature Fusion (CSFF) and Morphological Edge Suppression (MES) mechanisms, the proposed framework achieves precise geometric localization and quantitative evaluation of early-stage structural damages (e.g., fatigue micro-cracks and looseness). The main contributions of this paper are summarized as follows:

(1) Instead of introducing a new segmentation foundation model, we develop a SAM-guided structural isolation strategy (CSCSAM) tailored for UAV-based catenary inspection. By combining component localization information with SAM prompts, the proposed strategy effectively suppresses environmental and operational variations and provides clean structural ROIs for subsequent anomaly detection.
(2) Based on the normalizing flow framework, we propose an Asymmetrical Parallel Flow architecture with scale-aware flow-depth allocation. Unlike conventional symmetric flow models that use identical transformation depths across feature scales, the proposed design adaptively assigns computational capacity according to feature characteristics, improving anomaly modeling efficiency and reducing sensitivity to high-frequency noise.
(3) We design two task-specific enhancement mechanisms, including Cross-Scale Feature Fusion (CSFF) and Morphological Edge Suppression (MES), to improve pixel-level anomaly localization. CSFF integrates global structural semantics with local defect patterns, while MES reduces boundary-related false alarms.
(4) We construct CSCUD, a UAV-based catenary component anomaly detection dataset, providing a realistic benchmark for evaluating unsupervised structural health monitoring methods.

The rest of this paper is organized as follows. Section 2 introduces the proposed method. Section 3 describes the experimental datasets, experimental setup, experimental results, and analysis. Section 4 Discussion, Section 5 Conclusions.

## 2. The Proposed Method

**Figure 1:**
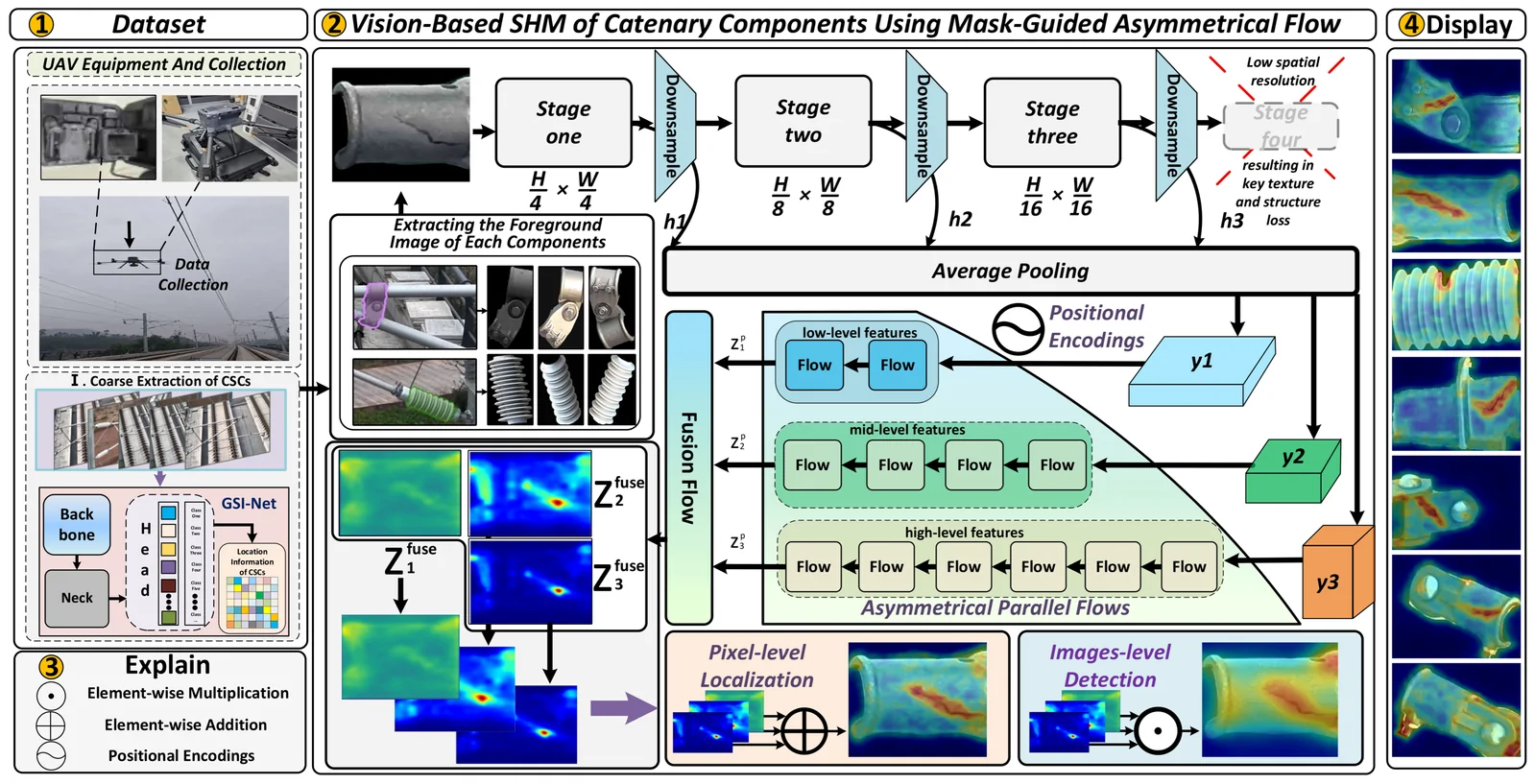
Architecture of the proposed MGAF framework. Following SAM-guided structural ROI extraction, a feature pyramid is constructed using a WideResNet-50 backbone. An Asymmetrical Parallel Flow architecture maps these multi-scale features into a latent space, while the subsequent Cross-Scale Feature Fusion (CSFF) module exchanges global semantic and local texture information to achieve precise pixel-level localization and image-level detection localization.

As illustrated in Figure 1, to achieve reliable unsupervised identification of multi-scale structural damages in railway catenary components while suppressing interference from complex Environmental and Operational Variations (EOVs), we propose a vision-based structural health monitoring framework named Mask-Guided Asymmetrical Flow (MGAF). The framework consists of three key modules: structural Region of Interest (ROI) extraction, healthy baseline probabilistic modeling via multi-scale flows, and mask-guided damage indicator quantification. The following sections elaborate on each component of the proposed framework.

Our methodology builds upon advanced multi-scale normalizing flow theory and incorporates an Asymmetrical Parallel Flow architecture alongside a Cross-Scale Fusion Flow mechanism. In addition, a novel edge-aware mask scoring strategy is introduced to ensure robust condition assessment for both macroscopic structural deformations and early-stage fatigue micro-cracks within a unified diagnostic pipeline.

The workflow begins with high-resolution aerial imagery of catenary systems acquired by unmanned aerial vehicles (UAVs) during field inspections. Specifically, structural bounding boxes are first extracted using a target detection algorithm (GSI-NET) [28] and then provided as spatial prompts to a segmentation foundation model (SAM). The bounding box annotations required for training GSI-NET were initially provided by experienced annotators. After obtaining reliable detection capability, GSI-NET was used to automatically generate component localization boxes for large-scale ROI extraction. The background regions are masked with black padding to explicitly isolate the structural components from environmental clutter, thereby constructing a clean, EOV-free dataset for establishing the healthy structural baseline. Subsequently, a multi-scale feature pyramid is extracted and passed through the flow-based model to map the normal features into a standardized latent space. During the condition assessment phase (inference), a mask-weighted Top-K scoring mechanism is applied to generate precise pixel-level damage localization heatmaps and image-level structural health classifications. It should be noted that GSI-Net and SAM are only utilized during the offline dataset preparation stage for structural ROI generation. They are not included in the online MGAF inference pipeline.

### 2.1. Overview of the MGAF Framework

In unsupervised anomaly detection, the core objective is to learn the probability distribution p(x) of normal samples Xnorm, such that anomalous samples Xanom fall into the low-density regions of the learned distribution. To enable accurate defect detection under the complex environmental conditions of railway catenary systems, we propose a Mask-Guided Asymmetrical Flow (MGAF) framework.

As illustrated in Figure 1, MGAF is a generative framework designed to map the complex distribution of high-resolution component images into a tractable latent distribution (e.g., a standard normal distribution) through a series of invertible transformations. Unlike traditional flow-based models that treat all image regions equally, MGAF incorporates a mask-guided mechanism that focuses density estimation exclusively on the relevant component areas, thereby completely filtering out background noise.

The MGAF framework consists of three tightly coupled stages:

(1) Multiscale Feature Pyramid Extraction: Given a preprocessed input image x ∈ ℝ*^H^^×W^^×C^*, where background regions have been masked to zero, extracting features from a single scale is insufficient for simultaneously capturing both macro-level structural anomalies and micro-level surface cracks. Therefore, we adopt a pre-trained WideResNet-50 as the backbone to construct a feature pyramid. Specifically, we extract feature maps from the first three stages, denoted as $\left\{h_{1},h_{2},h_{3}\right\}$. To balance computational efficiency and spatial resolution, each feature map is subjected to 3 × 3 average pooling with a stride of 2, resulting in the final multiscale inputs y = {y1, y2, y3}. Here, y1 preserves fine-grained textures for detecting minor cracks, while y3 encodes high-level semantic cues for identifying missing or deformed components.
(2) Asymmetrical Parallel Flows: A key innovation of MGAF is its asymmetrical parallel design. We observe that higher-level features (y3), with more channels and richer semantics, require deeper transformation networks to accurately model their distributions. In contrast, lower-level features (y1) have higher spatial resolution but simpler semantics. To address this heterogeneity, MGAF allocates different numbers of flow steps to each scale: fewer flow modules are applied to y1 to prevent overfitting and reduce computational overhead, while more modules are stacked for y3 to ensure sufficient representational capacity. This asymmetrical structure significantly improves inference speed, making the system suitable for real-time catenary inspection.
(3) Cross-Scale Integration for Contextual Damage Validation: While the asymmetrical parallel flows ensure the real-time processing efficiency required for online condition monitoring, they inherently process multi-scale structural features independently, which can lead to scale isolation. For instance, without macroscopic global context, benign local textural variations (e.g., surface weathering or harmless manufacturing marks) may be misclassified as early-stage structural damages.

To overcome this limitation, MGAF introduces a lightweight Fusion Flow module subsequent to the parallel flows. This module aggregates the latent representations of structural states from all scales, facilitates information exchange via global convolutions to capture the overall structural topology, and redistributes the context-aware features back to each respective scale. Such cross-scale interaction ensures that localized damage quantifications are contextually validated against the global structure, effectively minimizing false alarms triggered by local Environmental and Operational Variations (EOVs).

From a mathematical perspective, the entire MGAF process establishes a bijective mapping f:Y→Z, where Y represents the multiscale input features and Z denotes the corresponding latent variables. The final anomaly score is computed based on the negative log-likelihood (NLL) of the estimated latent variables Z, and is strictly constrained by the segmentation mask, ensuring that only foreground (component) regions contribute to the score while background interference is completely excluded.

In summary, the MGAF framework is not merely a probabilistic density estimation model, but a task-specific, vision-based structural health monitoring (SHM) system tailored for the complex Environmental and Operational Variations (EOVs) inherent in field-level condition assessments.

By synergistically integrating the robust structural ROI extraction capabilities of SAM with the fine-grained healthy baseline modeling power of multi-scale flow networks, MGAF effectively overcomes the longstanding performance bottleneck in traditional unsupervised methods between EOV suppression and cross-scale damage identification. This design enables MGAF to perform high-precision, full-spectrum structural condition assessments—ranging from macroscopic structural deformations (e.g., suspension fractures) to early-stage fatigue micro-cracks (e.g., insulator degradation)—without requiring any prior structural damage samples during training. As such, it offers a reliable, efficient, and real-time deployable non-destructive evaluation (NDE) solution for ensuring the structural integrity and operational safety of critical transportation infrastructure.

### 2.2. Segment Anything Model with Component Position In-Formation (CSCSAM)

**Figure 2:**
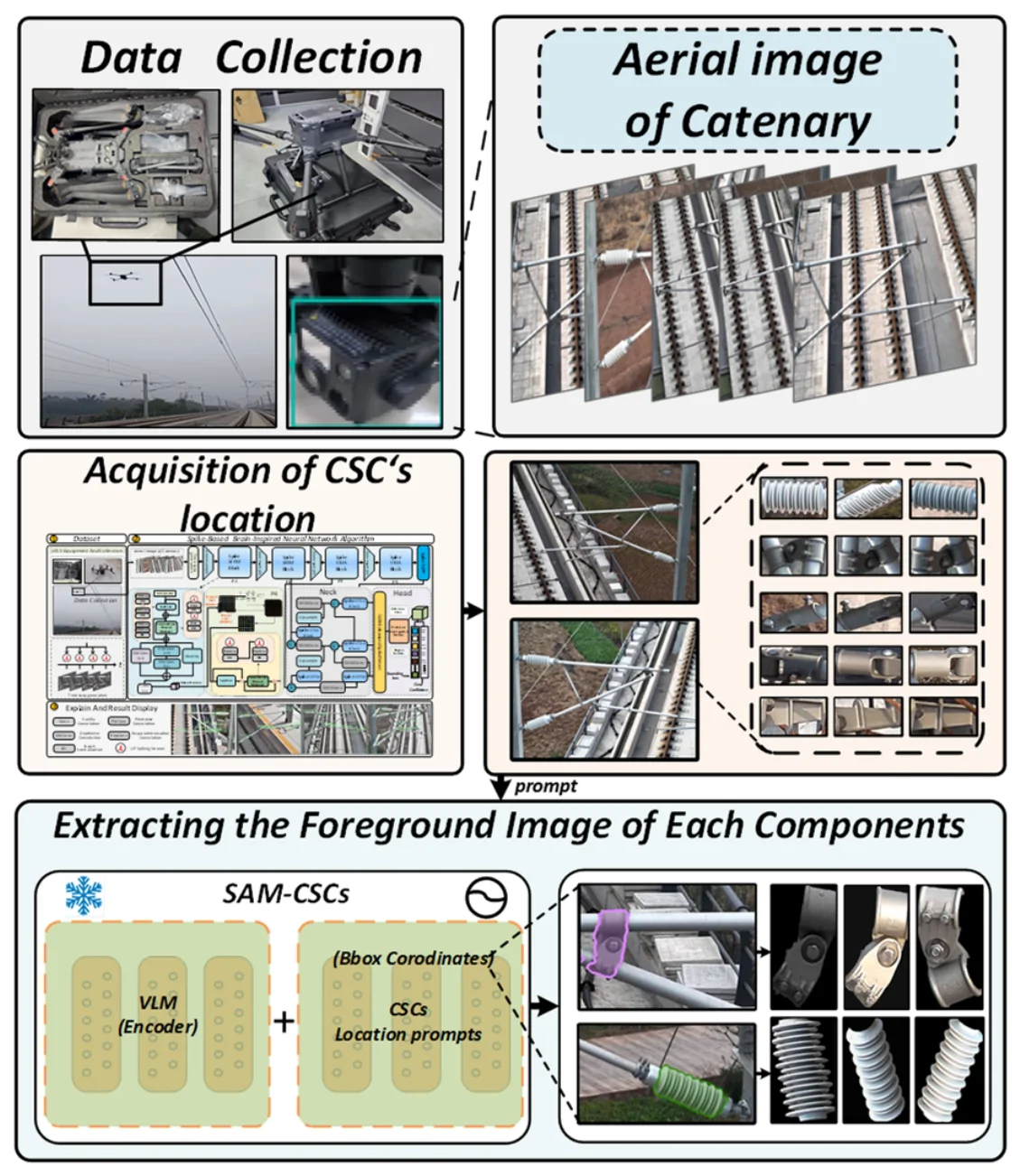
The flowchart for CSCSAM.

To enable precise localization and the elimination of Environmental and Operational Variations (EOVs) prior to structural damage identification in catenary components, we propose an adaptive structural Region of Interest (ROI) extraction pipeline (see Figure 2). This pipeline is specifically designed to address two inherent condition assessment challenges posed by UAV-acquired aerial imagery: First, UAVs must operate at safe altitudes of 50–100 m, resulting in extremely small image footprints for critical load-bearing components—especially fasteners and connectors. This significantly increases the difficulty of identifying early-stage structural damages, such as fatigue micro-cracks. Second, the top-down perspective of aerial imaging inevitably introduces severe EOVs (e.g., background clutter from ballast, tracks, and vegetation). It is imperative to explicitly suppress these environmental interferences beforehand to focus the diagnostic process exclusively on the effective structural ROIs.

Specifically, during the construction of the CSCUD dataset, the bounding boxes of catenary components were initially annotated by experienced researchers and used to train our previously developed GSI-NET detector. After training, GSI-NET was employed to automatically localize the structural components from high-resolution UAV images and generate bounding boxes for subsequent ROI extraction. Based on these automatically generated bounding boxes, each component region was extracted and provided as a spatial prompt to SAM for structural ROI segmentation. It should be noted that GSI-NET and SAM were mainly utilized during the dataset preparation stage to facilitate efficient component localization and structural region extraction. The pixel-level defect masks used for anomaly localization evaluation were manually annotated by experienced researchers to provide reliable ground-truth labels. GSI-NET adopts a three-level spiking neural network architecture consisting of a spatiotemporal feature extraction backbone, a multi-scale fusion neck, and a graph-guided detection head. Built upon the IB-LIF (Integer-valued training Binary inference Leaky Integrate-and-Fire) neuron model, it achieves sparse event-driven inference with low energy consumption. The detector is pre-trained on 3200 annotated UAV images covering all seven catenary component categories, with an input resolution of 640 × 640 pixels. It automatically outputs candidate bounding boxes, which are filtered by a confidence threshold of 0.7 and processed with non-maximum suppression (IoU threshold = 0.5) before being fed into SAM as spatial prompts. To reduce the additional burden of manually annotating structural ROI masks, we propose a SAM-based structural isolation scheme, we propose a SAM-based structural isolation scheme, termed CSCSAM. In this scheme, each UAV image and its corresponding bounding box are provided as spatial prompts to SAM [29], enabling the foundation model to infer high-precision structural masks. We use the official ViT-Base version of SAM (checkpoint: ‘sam_vit_b_01ec64.pth’, released by Meta AI) as the segmentation foundation model. All parameters of SAM remain frozen throughout the study without any fine-tuning on the catenary dataset. The output probability masks are resized to the original image resolution and binarized with a threshold of 0.5 to obtain the final structural foreground masks. It should be noted that GSI-NET provides only coarse structural localization information rather than defect-level annotations. Therefore, moderate bounding-box deviations mainly influence the ROI boundary instead of the internal structural representation. Benefiting from the prompt-based segmentation capability of SAM, the proposed CSCSAM strategy can further refine the component regions and alleviate the influence of localization uncertainty. Nevertheless, inaccurate bounding boxes may introduce additional background information or lose partial structural regions, which could negatively affect anomaly localization performance. Therefore, confidence filtering and non-maximum suppression are employed to ensure reliable spatial prompts. This process effectively decouples the mechanical components from the background EOVs, and is formally defined in Equation (1).

(1) $$Fg_{n}^{C}\;=SAM((Bbox_{n}^{c}\;,xi\;),\theta_{SAM}\;)$$

Here, ${Fg}_{n}^{C}$ denotes the extracted component foreground after segmentation, while SAM and $\theta_{\mathrm{SAM}}$ represent the segmentation model architecture and its fixed weight parameters, respectively. In this process, SAM’s image encoder ingests the panoramic aerial imagery, while the prompt encoder processes the spatial guidance information defining the geometric boundaries of the catenary components. This cascaded “localization-to-isolation” pipeline offers two significant advantages for practical structural health monitoring: First, it explicitly filters out the complex Environmental and Operational Variations (EOVs) inherent in UAV-captured imagery. This constructs a highly reliable, interference-free structural Region of Interest (ROI) foundation for subsequent unsupervised condition assessment. Second, it refines the diagnostic focus from a macroscopic infrastructural view down to a highly localized component perspective. This isolation mechanism substantially enhances the system’s sensitivity to early-stage structural damages (e.g., fatigue micro-cracks and looseness), which are otherwise easily obscured by environmental clutter.

### 2.3. MGAF Network Architecture

This section provides a detailed explanation of the core network components of the proposed MGAF framework. As illustrated in Figure 1, the architecture is designed to perform multi-scale probabilistic modeling, mapping the complex and heterogeneous feature distributions of catenary components into a tractable latent Gaussian space for effective and interpretable anomaly detection.

#### 2.3.1. Multi-Scale Feature Pyramid Extraction with Rich Hierarchical Semantics

In the task of railway catenary component anomaly detection, component defects exhibit extreme size variation. For instance, the complete fracture of a suspension rod or structural deformation of a positioning tube are categorized as macro-level structural anomalies, primarily characterized by geometric distortions. In contrast, micro-level texture anomalies—such as fine cracks on insulator surfaces or loosening of split pins—are manifested only in local high-frequency details. Conventional single-scale feature extraction often fails to strike a balance between these two extremes: deep-layer features offer strong semantics but suffer from low spatial resolution, leading to the loss of small cracks; meanwhile, shallow-layer features retain fine spatial details but have a limited receptive field, making it difficult to detect large-scale structural damage.

To overcome this contradiction, constructing a multi-scale feature pyramid with rich hierarchical semantics forms the foundation of the proposed MGAF framework.

Backbone Network and Feature Hierarchy Design: Given the complex backgrounds and diverse textures present in catenary component imagery, a feature extractor with strong representation capacity and wide-channel capability is essential. To this end, we adopt the WideResNet-50-2 (WRN-50-2), pre-trained on the ImageNet dataset, as our backbone network. Compared with standard ResNet, WideResNet increases channel width instead of depth, allowing it to more effectively capture fine-grained patterns in industrial images while mitigating gradient vanishing in deep networks. After SAM-based preprocessing and background removal, the input image is denoted as $x\in R^{H\times W\times C}$, where Hand Wrepresent the spatial resolution and C=3 corresponds to RGB channels. The image is fed into the backbone network, and intermediate feature maps are extracted from the first three main convolutional stages. We formalize the feature extraction process as a function Φ(⋅)

(2) $$H=\{h_{1},h_{2},h_{3}\}=\Phi_{WRN}(x)$$

Here, $h_{i}$ denotes the raw feature map output from the *i*-th stage of the backbone network. In WideResNet-50-2, these three stages produce semantically and structurally distinct features: h1 (Stage 1): Spatial resolution of H/4×W/4, with 256 channels. This layer is primarily responsive to edges, corners, and texture gradients, making it highly sensitive to local anomalies. h2 (Stage 2): Spatial resolution of H/8×W/8, with 512 channels. This layer begins to aggregate local patterns, forming mid-level component representations. h3 (Stage 3): Spatial resolution of H/16×W/16, with 1024 channels. This layer captures highly abstract semantic information and is particularly effective for identifying global structural anomalies. However, directly feeding the raw features hiinto the flow-based model presents two main challenges: (1) The high dimensionality of the feature maps results in excessive computational overhead. (2) The original convolutional features may retain undesirable high-frequency noise, which undermines the smoothness assumptions required for effective density estimation in normalizing flows. To address these issues, we propose a Dense Average Pooling strategy to construct the final feature pyramid. For each feature map hi, we apply average pooling with a kernel size of *k* = 3 and stride s=2. This operation is mathematically equivalent to low-pass filtering followed by downsampling, which effectively suppresses aliasing effects and enhances the robustness of the features. The resulting multi-scale feature pyramid $\left\{y_{1},y_{2},y_{3}\right\}$ is computed as follows:

(3) $$y_{i}={\mathrm{AvgPool}}_{k=3,s=2}(h_{i}),\;\;\;\;i\in \{1,2,3\}$$

Specifically, for each channel c and spatial location uv in the feature map hi, the output yi is computed as

(4) $$y_{i}^{\left(c\right)}(u,v)=\frac{1}{k^{2}}\sum_{m=0}^{k-1}\sum_{n=0}^{k-1}h_{i}^{\left(c\right)}(s\cdot u+m,s\cdot v+n)$$

After the transformations described above, we obtain the final multi-scale feature set $Y=\{y_{1},y_{2},y_{3}\}$. These features not only form a pyramid structure with spatial resolutions of H/8, H/16,H/32, but also provide comprehensive coverage across semantic dimensions, from “texture” to “structure”.

In the context of catenary component detection, this pyramid structure has a clear physical correspondence: y1 (Stage 1): Similar to a “microscope,” its high-resolution characteristics enable it to capture fine details on the component surface, such as micro-cracks. y2 (Stage 2): Similar to a “magnifying glass,” it detects medium-scale defects, such as partial damage or foreign objects on the components. y3 (Stage 3): Similar to a “telescope,” with a wide receptive field, it enables understanding of the topological structure of the components, thus identifying severe failures like missing components or large-scale deformations.

It is important to note that, although WideResNet-50 includes a fourth convolution stage (Stage 4), we intentionally exclude it from the feature pyramid in the MGAF framework for three key reasons: (1) Spatial Resolution Collapse: Unlike image classification, anomaly detection not only requires identifying “whether anomalies exist,” but also generating pixel-level heatmaps to localize “where anomalies occur.” The original feature map h4 from Stage 4 has a resolution of only 1/32 of the input image. After applying our dense pooling operation (stride s = 2), the resulting feature map y4 would have a resolution of just 1/64. For example, with an input image of size 256 × 256, y4 would be 4 × 4. At such a low resolution, spatial information is nearly lost, rendering it incapable of providing effective localization clues and even causing checkerboard artifacts during upsampling, which would blur anomaly boundaries. (2) Semantic Saturation and Over-Abstraction: The features from Stage 3 (y3) already have a sufficient receptive field to cover the entire catenary components (e.g., insulator strings or bases) and can capture global structural anomalies. The features extracted by Stage 4, however, tend to be overly abstract and biased towards class-specific semantics. For unsupervised anomaly detection, the “normal distribution density estimation” approach benefits from detailed, discriminative structural features, and excessive abstraction would filter out such information, leading to diminishing returns in terms of information gain. (3) Computational Efficiency Considerations: The number of channels in Stage 4 is typically as high as 2048. Including these features in the flow model would significantly increase the number of parameters and the computational load (FLOPs) in the affine coupling layers due to the 1 × 1 convolutions. By discarding Stage 4, we can focus computational resources on the first three scales, ensuring real-time performance on embedded inspection devices without compromising detection accuracy.

Through this well-designed multi-scale feature extraction mechanism, the MGAF framework guarantees the complete-ness of information at the input stage, laying a solid data foundation for the subsequent asymmetrical probabilistic modeling.

#### 2.3.2. Asymmetrical Parallel Flow Modeling

After obtaining the multi-scale feature pyramid $Y=\{y_{1},y_{2},y_{3}\}$, the core task of the MGAF framework is to learn the probability density functions $p(y_{i})$ of normal catenary components at each scale. To achieve this, we introduce the Normalizing Flow model. Normalizing flow maps complex feature distributions to simpler latent space distributions via a series of invertible transformations f. However, traditional flow models typically apply a uniform network depth (i.e., the same number of flow modules) across all scales. This uniform design is suboptimal for catenary detection tasks. Therefore, we propose an asymmetrical parallel flow architecture to address these limitations.

The three flow branches adopt different numbers of flow blocks, i.e., $K_{1}=2$, $K_{2}=4$, and $K_{3}=8$ for $y_{1},y_{2},y_{3}$, respectively. This progressive allocation follows the multi-scale flow design principle, where deeper transformations are assigned to higher-level semantic features with more complex distributions.

The design principle of asymmetric depth is based on the fact that features at different scales have distinct properties, requiring differentiated model capacity allocation. High-level semantic features (y3): With a high channel dimension C=1024 and complex semantic distributions, y3 encodes the overall topology of components (e.g., the concept of “insulator strings”). Due to its complex distribution, shallow flow models are insufficient to decouple it into a Gaussian distribution, leading to underfitting. Therefore, more flow modules (e.g., $K_{3}=8$) are stacked to ensure adequate non-linearity. Low-level texture features (y1): With a lower channel dimension C=256 and rich high-frequency spatial details, y1 has high resolution but relatively simple semantics (mainly edges and lighting). Using a deep network at this stage would incur exponential computational cost and lead to overfitting on the background noise. Hence, only a few flow modules (e.g., $K_{1}=2$) are used to meet density estimation requirements. Based on this analysis, MGAF constructs an independent flow network $f_{\Theta_{i}}$ for each scale i, with depth Kimonotonically increasing across scales. This asymmetric design cleverly balances model expressiveness and computational efficiency, which is key to enabling real-time, high-precision detection in MGAF.

**Figure 3:**
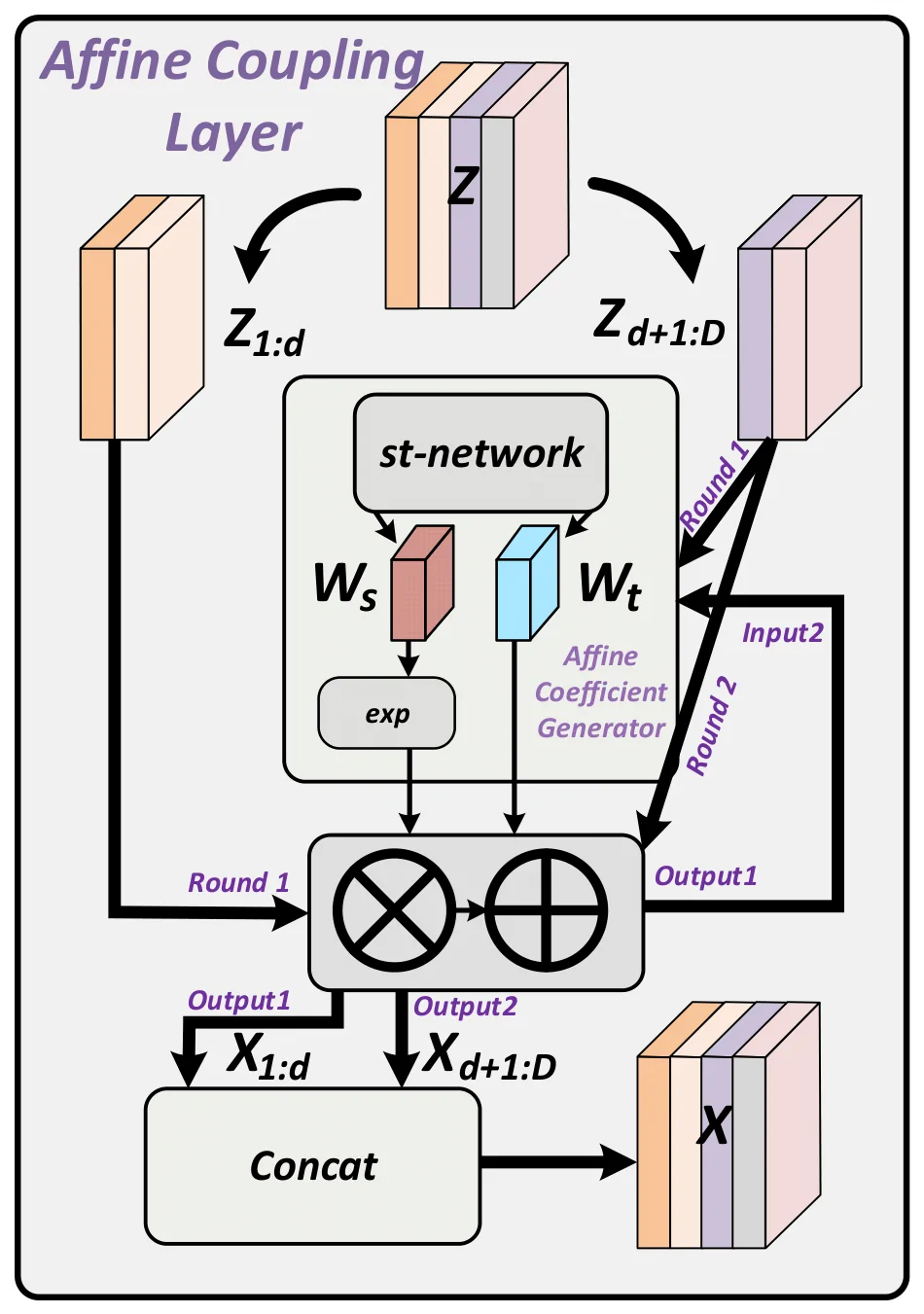
The flow block consists of two affine coupling layers that encode the split features, with the outputs being concatenated to produce the final result.

As illustrated in Figure 3, the mathematical mechanism of the affine coupling layer is as follows: Each flow network $f_{\Theta_{i}}$ consists of Kiinvertible affine coupling layers (ACLs) stacked in sequence. These layers are the core components for achieving invertible mappings and efficient computation of the Jacobian determinant. For the k-th flow module, the input is u (the output from the previous layer), and the output is v. We first split the input u along the channel dimension into two parts: u1:d and ud + 1:D, where d is the splitting point (usually set to half the number of channels). 

The affine coupling transformation is defined as

(5) $$\begin{array}{l} v_{1:d}=u_{1:d}\\ v_{d+1:D}=(u_{d+1:D}-t(u_{1:d}))⊙\exp(-s(u_{1:d})) \end{array}$$

where $v_{1:d}$ remains unchanged and serves as the “conditioning” information; $v_{d+1:D}$ undergoes a scaling and translation transformation. ⊙ denotes the Hadamard product (element-wise multiplication). s(⋅) and t(⋅) are the scaling factor and translation factor, respectively, fitted by a neural network. The introduction of the exponential function exp(⋅) ensures that the scaling factor remains positive, thereby guaranteeing the strict invertibility of the transformation. Inverse Transformation and Jacobian Determinant: Due to the special structure of this mechanism, the inverse transformation (used for generating samples from the latent space) is extremely simple

(6) $$u_{d+1:D}=v_{d+1:D}⊙\exp(s(v_{1:d}))+t(v_{1:d})$$

More importantly, the Jacobian matrix J = ∂v/∂u this transformation is a lower triangular matrix. Therefore, the logarithm of its determinant (used for calculating the Loss) only requires summing the diagonal elements, eliminating the need for expensive matrix operations

(7) $$u_{d+1:D}=v_{d+1:D}⊙\exp(s(v_{1:d}))+t(v_{1:d})$$

This enables MGAF to efficiently compute the likelihood probability on high-resolution feature maps.

In Equation (5), s(⋅) and t(⋅) represent the specific neural network, i.e., the st-network. In standard Real-NVP, these are typically implemented with fully connected layers or 1 × 1 convolutions, focusing on inter-channel correlations. However, catenary components are highly structured industrial products, and their anomalies are often closely related to their local spatial context (e.g., whether a nut exists depends on the surrounding bolt structure).

To adapt to this characteristic, we introduce two targeted improvements to the st-network: (1) 3 × 3 Convolution (Spatial Context Awareness): We replace the 1 × 1 convolution with a 3 × 3 convolution, which gives the flow model a receptive field. This allows the model to consider neighborhood features when computing the scaling and translation coefficients for a given position. This is crucial for distinguishing between normal texture fluctuations and micro-cracks. (2) Layer Normalization (LN): Industrial anomaly detection often faces memory limitations, resulting in smaller batch sizes during training. In such cases, Batch Normalization (BN) can lead to inaccurate statistical estimates, affecting model convergence. Therefore, we introduce Layer Normalization (LN) between the convolutional layers of the st-network, which normalizes independently of the batch dimension, significantly improving training stability. The improved st-network structure is represented as:

(8) $$st(x)={\mathrm{Conv}}_{3\times 3}(\mathrm{ReLU}(\mathrm{LN}({\mathrm{Conv}}_{3\times 3}(x))))$$

With this design, the asymmetrical parallel flow not only adapts to multi-scale features at the macro architecture level, but also perfectly aligns with the textural characteristics of catenary component images at the micro-component level.

#### 2.3.3. Cross-Scale Feature Fusion

Although the asymmetrical parallel flow effectively maps features yifrom different scales independently into the latent space, this “divide and conquer” strategy introduces a key theoretical flaw—scale isolation. In catenary anomaly detection, there is a strong dependency between local textures and global structure. For instance, certain normal lighting changes or dust accumulation on the surface of an insulator might appear as a high-contrast anomaly in y1 (the local high-frequency layer). However, when combined with information from y3 (the global semantic layer), it becomes clear that this is simply normal reflection from the insulator skirt’s edge, and the false positive can be excluded. Similarly, the texture of a nut might appear clear and complete in y1, but combining it with y3 reveals it is in the wrong topological location (e.g., a missing nut where one should be), indicating a severe structural failure. Specifically, the three feature representations extracted from WideResNet-50-2 have different spatial resolutions and channel dimensions. The corresponding feature maps are represented as:

(9) $$\begin{array}{l} y_{1}\in R^{H/4\times W/4\times 256},\\ y_{2}\in R^{H/8\times W/8\times 512},\\ y_{3}\in R^{H/16\times W/16\times 1024}. \end{array}$$

After passing through the independent flow branches, the latent features *z*_1_, *z*_2_, *z*_3_ are first spatially aligned using average pooling. The aligned features are then concatenated along the channel dimension:

(10) $$z_{cat}=Concat({\bar{z}}_{1},{\bar{z}}_{2},{\bar{z}}_{3})$$

where ${\bar{z}}_{i}$ denotes the spatially aligned feature representation. Subsequently, a convolutional fusion operation is applied to learn cross-scale correlations:

(11) $$z_{f}=Conv(z_{cat})$$

Finally, the fused representation is redistributed to each feature scale and combined with the original latent features:

(12) $$z_{i^{\prime }}=z_{i}+\phi_{i}(z_{f}),i=1,2,3$$

This cross-scale interaction enables high-level semantic information to guide the interpretation of local structural details, improving the localization capability of small defects.

If the flow models at different scales do not communicate, the model cannot leverage this contextual information. Therefore, after the parallel flows, MGAF introduces a Cross-Scale Fusion Flow module, aimed at establishing a unified joint probability distribution model.

The design objective of the Fusion Flow ffuse is to facilitate information exchange between latent variables at different scales. Let the set of latent variables from the parallel flows be $Z_{\mathrm{parallel}}=\{z_{1}^{p},z_{2}^{p},z_{3}^{p}\}$. The fusion process consists of three strict mathematical steps.

Spatial Unification & Concatenation: Since $z_{1}^{p},z_{2}^{p},z_{3}^{p}$ have different spatial resolutions (H/8,H/16,H/32 respectively), they cannot directly interact through channels. We employ an adaptive downsampling strategy to unify all latent variables to the smallest resolution (i.e., the size of $z_{3}^{p}$).

(13) $${\tilde{z}}_{i}=D(z_{i}^{p},\mathrm{size}(z_{3}^{p})),\;\;\;\;i\in \{1,2\}$$

Here, D(⋅) denotes the bilinear interpolation downsampling or average pooling operation. For $z_{3}^{p}$, which is already at the smallest size, it remains unchanged. Subsequently, the aligned features are concatenated along the channel dimension, forming a super-tensor $z_{\mathrm{cat}}$ that contains information from all scales:

(14) $$z_{cat}=\mathrm{Concat}({\tilde{z}}_{1},{\tilde{z}}_{2},{\tilde{z}}_{3})$$

The Global Interaction Transformation is the core of the fusion process. We input the concatenated $z_{\mathrm{cat}}$ tinto a lightweight flow module $f_{\mathrm{fuse}}$. This module simultaneously mixes the features across both channels and space using a 3 × 3 convolution kernel:

(15) $${z^{\prime }}_{cat}=g_{fuse}(z_{cat})$$

**Figure 4:**
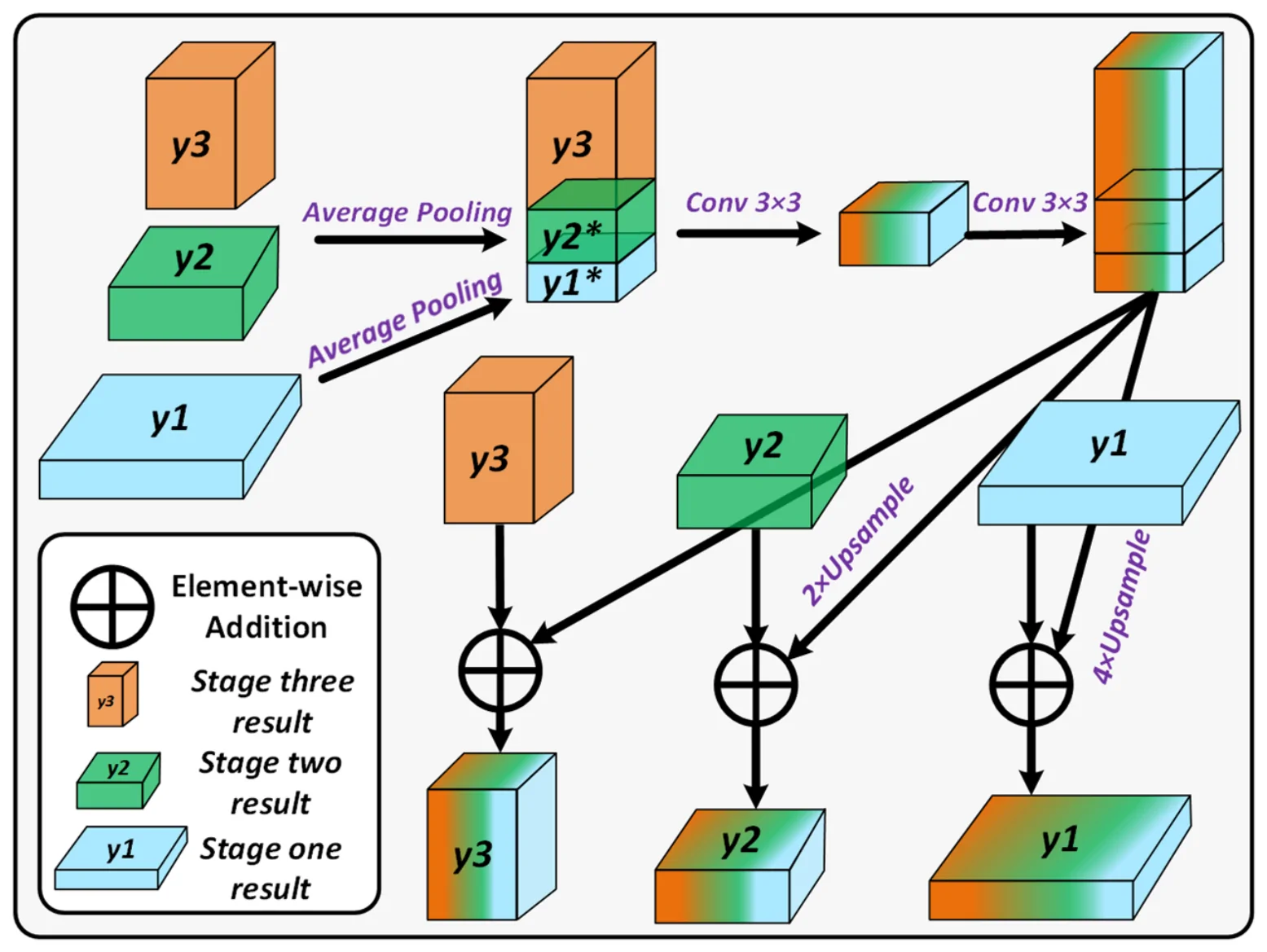
Detailed architectural design of the fusion network *g_fuse_* within the fusion flow’s st-network, where AP stands for average pooling.

As shown in Figure 4, in this process, low-level features (from $z_{1}^{p}$) are able to access high-level semantic information, while high-level features (from $z_{3}^{p}$) are able to perceive low-level texture details. $f_{\mathrm{fuse}}$ also employs an invertible affine coupling structure, ensuring the mathematical rigor of the overall framework.

Finally, after the Redistribution & Residual Correction interaction, we split the $z_{\mathrm{cat}}^{\prime }$ back into three parts $\left\{{\hat{z}}_{1},{\hat{z}}_{2},{\hat{z}}_{3}\right\}$, and use an upsampling operation U to restore each part to its original spatial resolution. The final fused latent variables $z_{\mathrm{fuse}}=\{z_{1\mathrm{fuse}},z_{2\mathrm{fuse}},z_{3\mathrm{fuse}}\}$ are generated through residual connections.

(16) $$z_{i}^{fuse}=z_{i}^{p}+\alpha_{i}\cdot U({\hat{z}}_{i},\mathrm{size}(z_{i}^{p}))$$

where $\alpha_{i}$ is a learnable weighting coefficient (initialized to a small value). This residual design ensures that the fusion flow serves only as a “correction” and “fine-tuning” of the parallel flow results, thereby accelerating the convergence stability of the model.

From a probabilistic perspective, the parallel flow only models the product of marginal distributions $\prod p(y_{i})$, assuming independence between scales. However, by introducing the fusion flow, MGAF is effectively modeling the joint distribution $p(y_{1},y_{2},y_{3})$. The total transformation of the entire MGAF network can be expressed as the composite function $F=f_{\mathrm{fuse}}\cdot f_{\mathrm{parallel}}$. According to the Change of Variables Formula, the final log-likelihood loss function Lis given by

(17) $$\begin{array}{l} L(Y)=\sum_{i=1}^{3}\left(\frac{1}{2}\|z_{i}^{fuse}{\|}_{2}^{2}\right)-L_{2}\\ L_{2}=log|\det J_{parallel}|+\log|\det J_{fuse}| \end{array}$$

The first term enforces that the fused latent variables strictly conform to a standard normal distribution, which mathematically represents the healthy baseline state of the structural components. The second term is the logarithm of the Jacobian determinant derived from the parallel flows, capturing the spatial geometric correlations of the structural topologies within each individual scale. The third term accounts for the additional volume change introduced by the fusion flow. Functioning as a contextual “correction factor,” this term explicitly models the cross-scale structural dependencies. It enables the model to dynamically calibrate the condition assessment of localized damage indicators based on the macroscopic structural context. Consequently, this formulation mathematically ensures the completeness and robustness of the structural integrity evaluation, effectively preventing local Environmental and Operational Variations (EOVs) from being mathematically misjudged as structural faults.

In practical railway catenary inspection scenarios, the cross-scale fusion flow acts as a “Contextual Verifier”, addressing two common detection challenges in traditional methods: (1) Suppressing Texture False Positives: The surface of catenary insulators often exhibits water stains, dust, or strong light reflections. In the low-level features y1 (high-resolution layer), these areas may present sharp texture transitions, easily misinterpreted as “cracks”. The fusion flow introduces global semantic information from the y3 layer. The model recognizes through y3 that this region is part of the smooth surface of the insulator skirt, with an intact geometric structure. The fusion flow then suppresses the anomaly response in y1 through weight adjustments in a 1 × 1 convolution, recalibrating the high-frequency signals as “normal lighting changes”, thus significantly reducing false positives. (2) Enhancing Structural Absence Detection: The absence of certain small fasteners (e.g., split pins or nuts) poses a significant safety hazard. However, from the local perspective of y1, the missing parts may only appear as a plain black background or hole, with no inherently “anomalous” texture features. The fusion flow injects topological structural information from y3. The model, by assessing the global structure, determines that a connection node should be present. Once it detects the lack of expected metallic texture in y1, a strong prediction error arises between the semantic prediction from y3 and the actual observation in y1. The fusion flow amplifies this cross-scale consistency error, significantly increasing the anomaly score for that region, ensuring that even subtle but critical structural absences are detected.

In summary, the fusion flow endows MGAF with holistic structural awareness, enabling the real-time contextual validation of microscopic damage indicators against macroscopic structural topologies. This capability is crucial for ensuring the structural integrity and operational safety of critical railway infrastructure.

### 2.4. Mask-Guided Training and Inference Strategy

Due to the SAM-based isolation of structural ROIs, the images input into the MGAF framework inevitably contain substantial masked background regions (zero-padding). Directly applying the standard flow-based probabilistic modeling paradigm to these partially masked inputs would introduce significant distribution bias into the healthy structural baseline and trigger severe false alarms at the structural boundaries. To address this algorithmic challenge, we developed a comprehensive mask-guided evaluation strategy. This strategy incorporates spatial optimization constraints during the baseline modeling (training) phase and morphological boundary suppression during the condition assessment (inference) phase, ensuring high-fidelity damage quantification.

#### 2.4.1. Mask-Constrained Training Objective

In standard unsupervised structural condition assessment, the optimization objective of flow-based models is typically to minimize the negative log-likelihood (NLL) over the entire spatial domain of the inspection imagery. However, in our preprocessed inputs, the environmental background is explicitly masked to zero to eliminate Environmental and Operational Variations (EOVs). If the loss function is computed over the entire spatial domain, the generative flow model would be forced to allocate disproportionate representational capacity to fit this artificial “Dirac delta distribution” (i.e., the zero-padded non-structural regions). This mathematical misalignment leads to two severe diagnostic consequences: (1) Gradient Dominance of Non-Structural Regions: The masked environmental background typically occupies more than 70% of the aerial field of view. The gradients generated by these zero-value regions would dominate the backpropagation process, causing the model to neglect the subtle damage indicators (e.g., fatigue micro-cracks) within the critical load-bearing components. (2) Distortion of the Healthy Baseline: The model would erroneously concentrate the probability density peak at the zero-value domain, pushing the actual healthy structural features into low-density regions. This distribution distortion inevitably leads to severe false alarms, where structurally sound components are mathematically misdiagnosed as degraded or faulty.

To resolve these mathematical misalignments and ensure a robust condition assessment, we propose a Mask-Constrained Loss formulation. This function utilizes the multi-scale binary masks $M=\{m_{1},m_{2},m_{3}\}$ generated by SAM as spatial attention gating. For the i-th scale feature yi and its corresponding mask mi (downsampled to the same resolution), we first define the local NLL loss at position $\left(u,v\right)$ as $l_{i}(u,v)$

(18) $$l_{i}(u,v)=\frac{1}{2}\|z_{i}^{fuse}(u,v){\|}_{2}^{2}-\log\left|\det\frac{\partial z_{i}^{fuse}}{\partial y_{i}}(u,v)\right|$$

The first term represents the Gaussian distribution constraint, while the second term represents the Jacobian determinant (volume change) constraint. The total loss function L total is defined as the sum of the average loss over all valid regions across scales

(19) $$L_{total}=\sum_{i=1}^{3}\frac{1}{N_{valid}^{\left(i\right)}}\sum_{u=1}^{H_{i}}\sum_{v=1}^{W_{i}}m_{i}^{\left(u,v\right)}\cdot l_{i}(u,v)$$

The normalization factor N(i) valid in the formula is crucial

(20) $$N_{valid}^{\left(i\right)}=\sum_{u,v}m_{i}^{\left(u,v\right)}+\epsilon$$

Unlike traditional methods that use a fixed total pixel count $H_{i}\times W_{i}$ for normalization, we use the number of valid foreground pixels. In catenary inspection, variations in the shooting distance cause the proportion of components in the image to fluctuate. By normalizing with $N_{\mathrm{valid}}$, we ensure that the gradient update strength remains constant regardless of the distance of the component (i.e., the size of the mask area), thus achieving scale invariance during training.

#### 2.4.2. Boundary-Aware Inference

During the inference phase, directly outputting the anomaly map often results in highlighted “pseudo-anomaly rings” at the boundaries between components and the background. Although SAM achieves high segmentation accuracy, the sharp transition from “complex component textures” to “pure black background” creates a high-frequency step signal. As a continuous probability density estimator, the flow model is mathematically challenging to perfectly fit such discontinuous step changes. Therefore, at the segmentation boundaries, the latent variable z often fails to fully converge to zero, resulting in a large norm $||z|{|}_{2}^{2}$, leading to false positives. To eliminate this systematic error, we introduce a morphology-based post-processing mechanism. First, we generate the raw anomaly map $A_{\mathrm{raw}}$ based on the maximum likelihood principle

(21) $$A_{raw}(u,v)=\sum_{i=1}^{3}U_{bilinear}\left(\|z_{i}^{fuse}(u,v){\|}_{2}^{2}\right)$$

Subsequently, we perform a morphological erosion operation on the original SAM mask M, generating the eroded mask $M_{\mathrm{eroded}}$

(22) $$M_{eroded}=M⊖K_{struct},\;\;\;\;K_{struct}\in {\mathbb{R}}^{5\times 5}$$

The final anomaly map $S_{\mathrm{final}}$ is obtained by filtering with the eroded mask $M_{\mathrm{eroded}}$

(23) $$S_{final}=A_{raw}⊙M_{eroded}$$

This operation is equivalent to “shrinking” the segmentation boundary by 2–3 pixels inward, cleverly avoiding the fitting blind spot of the flow model, while retaining over 95% of the component’s main region for detection.

#### 2.4.3. Top-K Global Scoring

To achieve image-level structural condition classification (i.e., “healthy” vs. “damaged”), the pixel-level damage localization heatmap must be aggregated into a global structural health indicator. Early-stage structural damages in catenary assemblies typically exhibit high mechanical criticality but occupy a minuscule proportion of the spatial structural ROI. For instance, the absence of a securing split pin might account for less than 0.1% of the total component area, yet it represents a severe threat to the overall structural integrity. Relying on conventional average pooling would erroneously dilute these critical, highly localized damage indicators with the vast majority of healthy structural features. Conversely, global max pooling is highly susceptible to false alarms triggered by isolated sensor artifacts or residual Environmental and Operational Variations (EOVs). Therefore, the MGAF framework adopts a robust Top-K pooling strategy to quantify the degradation severity.

(24) $$s_{img}=\frac{1}{\left|\Omega_{K}\right|}\sum_{j\in \Omega_{K}}S_{final}^{\left(j\right)}$$

where $\Omega_{K}$ represents the set of the top K highest-scoring pixels in the anomaly map $S_{\mathrm{final}}$, where Kis set to 1% of the total valid foreground pixels.

This aggregation strategy emulates the diagnostic intuition of a seasoned structural engineer. It autonomously bypasses macroscopically intact regions, concentrating analytical focus exclusively on highly localized damage indicators. Even if critical structural degradation (e.g., fatigue micro-cracks or fastener yielding) is confined to a minuscule fraction (e.g., less than 1%) of the component’s spatial domain, the system reliably classifies the overall structural condition as degraded, thereby providing a highly sensitive early warning against potential catastrophic failures.

## 3. Results

### 3.1. Experimental Settings

This study is grounded in real-world structural health monitoring (SHM) scenarios for railway catenary systems. To validate the engineering effectiveness of the proposed non-destructive evaluation (NDE) framework, we established a field-acquired dataset, designated as CSCUD. The field data acquisition was executed entirely using a DJI Matrice 350RTK unmanned aerial vehicle (UAV) (DJI, Shenzhen, China)., strictly navigating along the planned inspection flight paths of the railway infrastructure.

**Figure 5:**
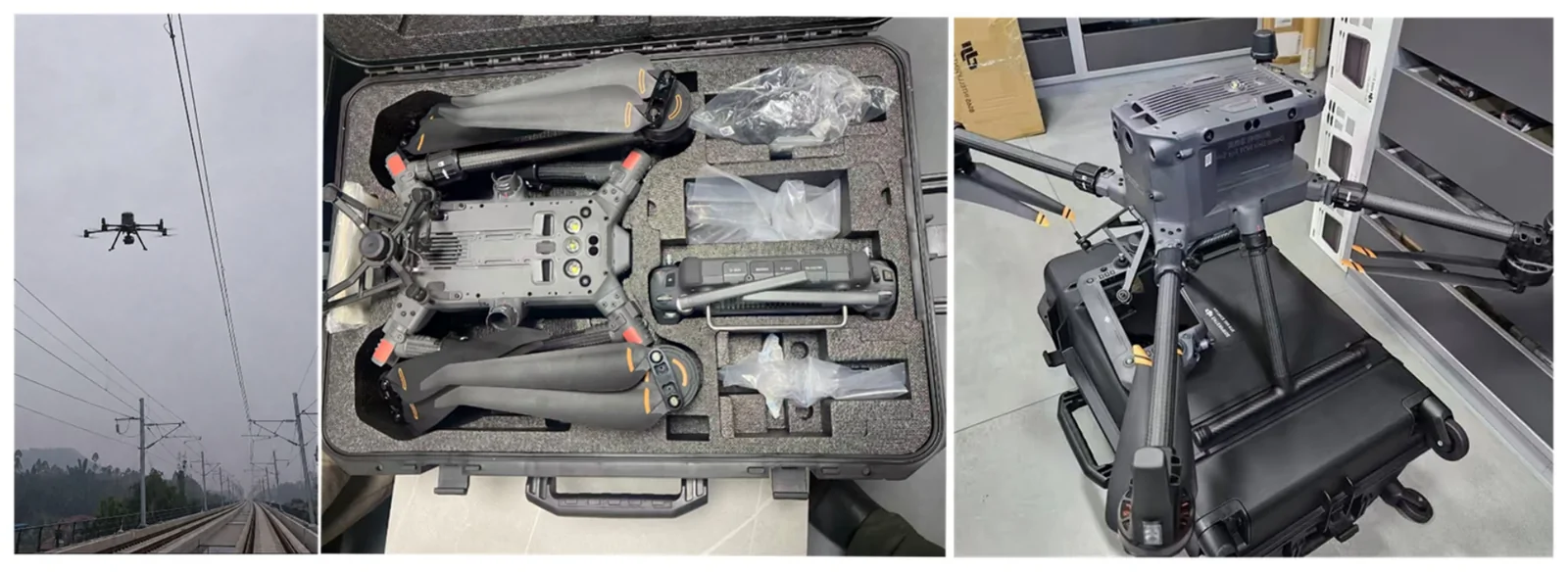
Aerial photography equipment for unmanned aerial vehicles.

To construct high-fidelity samples for establishing the healthy structural baseline, we employ a cascaded “localization-to-isolation” strategy to preprocess the raw aerial imagery. By integrating the GSINet detector with the SAM [25] foundation model, this strategy efficiently isolates the critical load-bearing components and explicitly suppresses complex Environmental and Operational Variations (EOVs). The resulting EOV-free structural Regions of Interest (ROIs) serve as the foundation for subsequent fine-grained structural damage identification tasks. The experimental evaluations focus on seven critical catenary support components that historically exhibit high rates of structural degradation. TThe sample distributions and UAV acquisition platform are presented in Table 1 and Figure 5, respectively.

Dataset Partition Strategy: Considering that UAV-based railway inspection images are usually collected through continuous flight trajectories, directly performing image-level splitting may introduce potential data leakage due to the high visual correlation among adjacent frames. Therefore, to ensure a reliable and unbiased evaluation, the CSCUD dataset was partitioned according to independent UAV acquisition sequences rather than individual images.

Specifically, images captured from the same railway line segment, UAV flight mission, or continuous video sequence were strictly assigned to only one subset. Thus, adjacent frames and visually similar samples from the same inspection trajectory were prevented from appearing simultaneously in the training and testing sets. This sequence-level partition strategy ensures that the proposed framework learns generalizable structural characteristics of catenary components rather than scene-specific visual patterns.

As an unsupervised anomaly detection framework, MGAF only utilizes normal samples during the training phase to establish the healthy feature distribution of catenary components. During the testing phase, both normal and defective samples are used to evaluate anomaly detection and localization performance. The final CSCUD dataset contains 10,577 normal images for training and 979 normal images together with 2272 abnormal images for testing across seven catenary component categories.

This partition protocol provides a more realistic evaluation scenario for practical UAV-based railway inspection, where the model is required to identify structural anomalies under previously unseen inspection conditions.

To ensure a fair and consistent comparison, all competing methods were evaluated using exactly the same CSCUAV dataset, which was constructed through the identical data processing pipeline. Specifically, GSI-NET and SAM were only used during the dataset construction stage to locate catenary components, extract structural regions, and generate standardized input samples. After dataset preparation, all anomaly detection methods, including MGAF and the compared baselines, received the same processed component images for training and evaluation. For all experiments, the same training/testing split, image preprocessing procedures, input resolution, and evaluation metrics were adopted for every method. The competing approaches were implemented according to their original papers with their default configurations and recommended hyperparameters, without introducing additional modifications except necessary adaptations to the dataset format. Therefore, the performance differences among different methods mainly reflect the effectiveness of their anomaly detection architectures rather than differences in data preparation. In this work, the official pre-trained SAM model is adopted without additional fine-tuning, and all SAM parameters remain frozen. The generated masks are only used as structural guidance to suppress irrelevant background information. Before being used in MGAF, the obtained masks are manually inspected to ensure that the major regions of catenary components are correctly segmented. Since the masks serve as intermediate ROI guidance rather than defect annotations, no additional pixel-level segmentation evaluation is performed.

Implementation Details: The experiments were conducted on a workstation equipped with an Intel Core i9-14900K processor and an NVIDIA RTX 4090 GPU. The environment was configured with CUDA 12.3 and CUDNN 8.9.7. The deep learning framework used was PyTorch 2.0.0 under Python 3.8. Both the GSINet and SAM modules were trained and evaluated using their default hyperparameter settings. For the GSI-NET detector, the SGD optimizer is adopted with an initial learning rate of 0.003, a momentum of 0.937, and a weight decay of 0.0005. The model is trained for 1250 epochs with a batch size of 2, and the bottom convolutional layers of the backbone are frozen for the first 100 epochs to preserve low-level feature representations. A cosine annealing learning rate scheduler with a 10-epoch linear warmup phase is applied to stabilize the membrane potential accumulation of spiking neurons in early training.

The main network configuration of the MGAF detector is as follows: To accommodate the multi-scale feature modeling needs of catenary components, we configured the hyperparameters of the feature extractor and asymmetrical flow generation network as follows: (1) Input Size: The input images during both training and inference are preprocessed using SAM and then resized to 256 × 256. (2) Backbone: The WideResNet-50-2, pre-trained on ImageNet, is used as the backbone network. During training, its parameters are frozen to prevent catastrophic forgetting. (3) Feature Pyramid: The output features from Stage 1, 2, and 3 are selected, with the feature channel dimensions being 2565121024, corresponding to three scale levels. (4) Asymmetrical Flow Depths: Considering the non-linearity of high-level semantics and the high-frequency nature of low-level textures, the number of flow blocks for each scale is configured asymmetrically as K=[4,8,8]. (5) Sub-network Configuration: Inside the affine coupling layers, a 3 × 3 convolution is used to expand the local receptive field, with 512 hidden channels. A soft-clamp (α = 1.9) mechanism is introduced to ensure numerical stability. (6) Fusion Flow: The cross-scale interaction module includes 2 global flow steps for unifying the multi-scale latent variable distributions.

Training Strategy: (1) Optimizer: The Adam optimizer is used, with an initial learning rate of 2 × 10^−4^, a weight decay of 1 × 10^−5^, and Betas parameters set to 0.50.9. (2) Learning Rate Scheduling: A cosine annealing strategy is applied, with the learning rate smoothly decaying to 0 over the entire training period, without a warm-up phase. (3) Iteration Settings: The model is trained for a total of 100 epochs, with a batch size of 16. (4) Data Augmentation: To maintain the purity of the normal sample distribution, geometrical distortion-based augmentations are not used. Instead, only numerical normalization (scaling to the [0, 1] range) and background zeroing based on the SAM mask are applied.

Finally, the model performance is evaluated using Average Precision (AP), Area Under the Receiver Operating Characteristic Curve (AUROC), and the F1 score.

### 3.2. Experimental Results and Analysis

To comprehensively validate the effectiveness of the proposed MGAF framework in real-world catenary inspection scenarios, we conducted extensive comparative experiments on the CSCUD dataset, which includes seven types of typical components (C1–C7). The quantitative results presented in Table 2 and Table 3 fully demonstrate the superior performance of the method in multi-class anomaly detection and localization.

#### 3.2.1. Performance on Multi-Class Anomaly Detection and Localization

In the image-level detection task, MGAF achieved optimal average performance with Mean Img-AUROC = 98.9%, Img-AP = 97.6%, and Img-F1 = 98.1%. Compared to MambaAD (based on state-space models) and Dinomaly (based on diffusion models), its F1-Score improved by 0.5% and 0.6%, respectively. Particularly for categories with relatively fixed structures, such as C1 (casing base), C2 (connection casing), and C3 (insulator), the asymmetrical parallel flow architecture effectively extracted the global topological structure in deep features (Stage 3, K = 8), achieving nearly perfect detection (with multiple metrics reaching 100% or 99.9%). This demonstrates the model’s ability to build high-discriminative boundaries under sample imbalance.

As shown in Table 4, in the more challenging pixel-level localization task, MGAF showed significant advantages, achieving Pixel-AP = 40.1% and Pixel-F1 = 56.7%. Compared to the second-best Dinomaly (Pixel-AP = 35.6%) and MambaAD (Pixel-AP = 29.6%), MGAF maintained a robust lead in localization accuracy, particularly improving by approximately 10.5 percentage points over MambaAD. This performance boost is primarily attributed to the cross-scale feature fusion flow, which injects high-level semantics into low-level features to ensure precise localization of micro-cracks, and the mask-guided training strategy, which suppresses background gradients. This effectively overcomes the limitations of traditional methods (e.g., SimpleNet), which generate blurred residual maps due to feature resolution loss.

To further evaluate the statistical reliability of the experimental results, 95% confidence intervals (CIs) were calculated based on the ten independent runs using Student’s t-distribution. For MGAF, the 95% CIs of I-AUROC, I-AP, I-F1, P-AUROC, P-AP, and P-F1 were [98.83, 98.97], [97.46, 97.74], [98.03, 98.17], [96.23, 96.37], [39.96, 40.24], and [56.49, 56.91], respectively. These relatively narrow confidence intervals indicate that MGAF maintains stable performance across repeated runs. In addition, statistical significance was assessed using two-sided paired *t*-tests between MGAF and the strongest competing method for each evaluation metric, with a significance level of *p* < 0.05. The statistical tests were conducted using the results from the same ten independent runs to ensure a consistent comparison protocol.

Robustness analysis for complex structures revealed that, even in the C3 (insulator) category, which has the most complex textures and is most affected by lighting, MGAF still maintained a Pixel-AP of 65.5% and Pixel-F1 of 59.9%, significantly outperforming RD4AD (39.4%) and MambaAD (36.7%), both of which experienced performance collapse. Meanwhile, in the C7 (double ear casing) category, which had the largest number of training samples, MGAF’s performance (Pixel-AP = 39%) also significantly exceeded SimpleNet (15.4%) and MambaAD (15.3%). This further proves that the morphological edge suppression mechanism, by eliminating common edge false alarms, endows the model with outstanding robustness and stability under large-scale data and complex texture interference.

#### 3.2.2. Comparative Summary of Different Detectors

From the results presented in Table 2 and Table 3, we can conclude that while methods based on emerging architectures (e.g., SSM or Diffusion) such as MambaAD and Dinomaly demonstrate outstanding performance in image-level classification due to their powerful generative capabilities, they still face limitations in tasks requiring pixel-level precision for defect segmentation (localization). This is particularly evident when handling fine components like C4 and C6, where resolution limitations or background interference often lead to a decline in accuracy.

In contrast, the MGAF framework, by introducing SAM-assisted background removal and multi-scale flow models for joint probability modeling, not only surpasses existing industrial anomaly detection methods in overall metrics, but more importantly, achieves high-precision pixel-level localization. It successfully generates defect heatmaps with clear edges and intact structures, effectively addressing common issues in traditional flow models such as edge artifacts and localization drift, demonstrating its great potential as the next-generation core algorithm for precision catenary inspection.

#### 3.2.3. Complexity Analysis

In addition to detection accuracy, computational overhead and inference latency are key metrics for evaluating whether an algorithm can be deployed in industrial settings. Table 5 provides a detailed comparison of MGAF with current SOTA methods in terms of computational efficiency (FLOPs, Params) and inference speed (Times).

(1) Superior Inference Efficiency via Single-Step Mapping

As shown in Table 5, MGAF achieves the best balance between accuracy and speed among unsupervised anomaly detection methods. Comparison with Diffusion Models: Although methods based on diffusion models, such as Dinomaly and DiAD, perform well in terms of accuracy (with Dinomaly achieving mAD = 79.8%), their inherent multi-step iterative denoising mechanism results in high time costs. Specifically, Dinomaly takes 0.155 s, while DiAD requires as much as 1.125 s.

Advantages of MGAF: In contrast, thanks to the single-step mapping mechanism of Normalizing Flows, MGAF can compute the likelihood probability with a single forward pass. This allows MGAF to not only surpass Dinomaly in detection accuracy (81.3% vs. 79.8%), but also achieve a 2× faster inference speed (0.077 s vs. 0.155 s), while reducing FLOPs by approximately 37% (65.9 G vs. 104.9 G). This “high accuracy, low latency” characteristic makes MGAF more suitable for deployment on edge computing devices with limited computational resources.

(2) Trade-off Between Capacity and Latency

Compared to lightweight models such as MambaAD (0.052 s) and SimpleNet (0.061 s), MGAF has a slightly higher inference time (approximately 10–25 ms longer). However, this minor delay results in a significant performance improvement: MGAF’s mAD is 4.0% and 2.8% higher than SimpleNet and MambaAD, respectively. Considering that MGAF’s single-frame inference time is 0.077 s (approximately 13 FPS), it still fully meets the typical 10 FPS acquisition frequency required for catenary inspection vehicles. For railway safety scenarios that are highly sensitive to false negative rates, sacrificing millisecond-level speed for higher reliability is a highly valuable engineering trade-off.

(3) Analysis of Model Complexity

The MGAF model has 216.5 M parameters. This is primarily due to the use of the wide-channel WideResNet-50 as the backbone network and the stacking of deep flow modules on high-level semantic features (Stage 3) to capture complex topological structures. It is worth noting that since the backbone network is frozen during training, the actual number of trainable parameters is much smaller, ensuring that training remains efficient.

Despite the large number of parameters, MGAF’s FLOPs are controlled at 65.9 G. This is mainly attributed to the proposed asymmetrical architecture: we configure only a small number of flow modules in the high-resolution Stage 1, thus avoiding costly dense convolution operations on millions of pixels. This allows MGAF to retain the feature extraction capability of a large capacity model without incurring an explosive computational burden.

#### 3.2.4. Visualization and Analysis of Anomaly Detection Results

**Figure 6:**
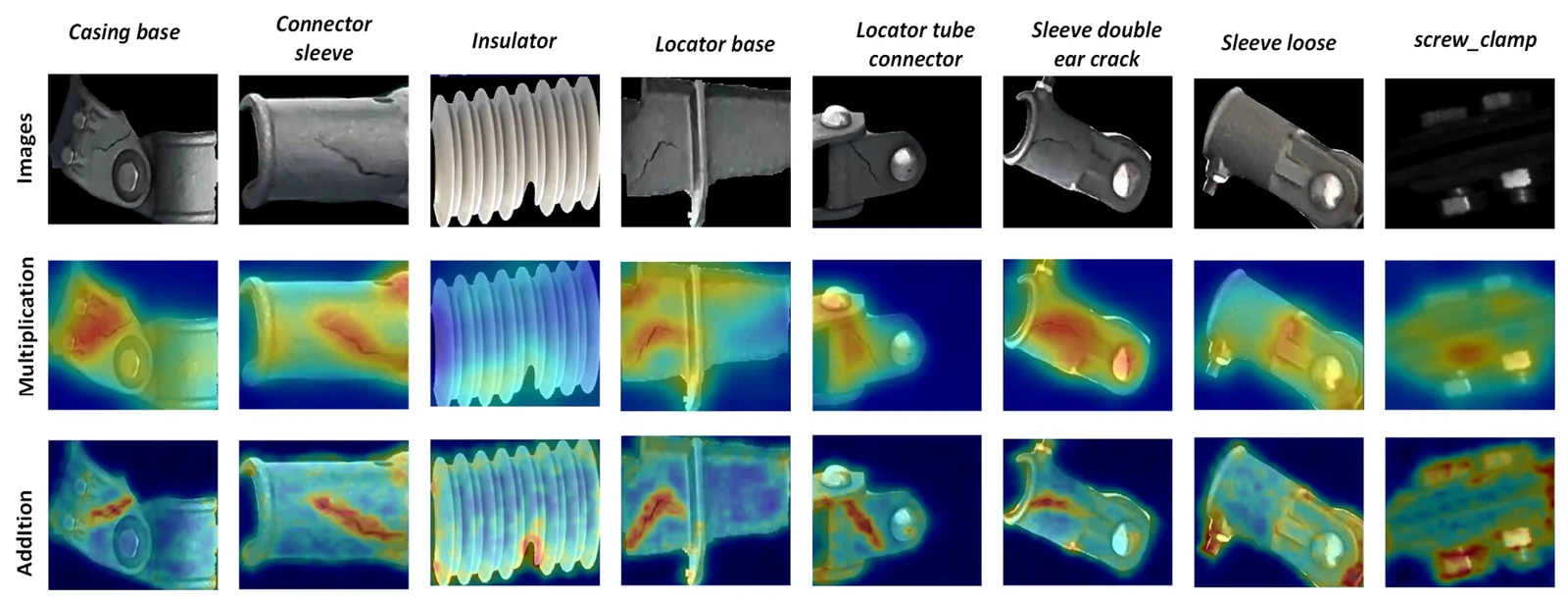
Component Fault Detection Results—Figure Explanation: The first row of Figure 6 displays the original input images containing real defects, such as surface cracks and fastener looseness. The second row shows the anomaly score maps generated using the Multiplication Fusion strategy. The third row presents the final results generated using the Addition Fusion strategy employed in this paper.

To further validate the engineering effectiveness of the MGAF framework from a qualitative condition assessment perspective, and to explore the impact of different cross-scale integration strategies on the reliability of damage localization, we conducted a detailed visualization analysis, as illustrated in Figure 6. This figure presents a comparative evaluation of damage localization heatmaps for several critical catenary components under different cross-scale fusion paradigms.

Comparison of Integration Strategies: By comparing the second and third rows, it is evident that the multiplicative fusion strategy frequently leads to the “over-suppression” of critical damage indicators. Due to the extreme mathematical sensitivity of the multiplication operation, if a specific scale (e.g., the macroscopic, low-resolution Llow) fails to exhibit a strong response to highly localized early-stage damages, the overall structural health indicator score is forcibly attenuated. This results in fragmented damage heatmaps and even the complete missed detection of critical fatigue cracks. In contrast, the third row of Figure 6 demonstrates that the additive integration strategy effectively accumulates structural degradation evidence across different scales (Constructive Accumulation). Whether it involves the localized damage indicators (e.g., surface micro-cracks) captured by the high-resolution Lhigh layer or the macroscopic structural deformations (e.g., missing components) identified by the low-resolution Llow layer, both are robustly preserved and enhanced in the final diagnostic heatmap.

The structural health indicator maps generated by the additive integration strategy proposed in this paper not only exhibit robust suppression of Environmental and Operational Variations (EOVs) (i.e., clean backgrounds), but also precisely localize and highlight early-stage structural damages in various catenary assemblies, including fatigue crack propagation and hidden fastener looseness. The clear geometric boundaries and complete damage morphologies strongly demonstrate the robustness and engineering applicability of MGAF in handling complex field-induced structural damages, validating its significant potential as a high-performance, vision-based non-destructive evaluation (NDE) diagnostic tool.

#### 3.2.5. Validation of Foreground Extraction Effectiveness

**Figure 7:**
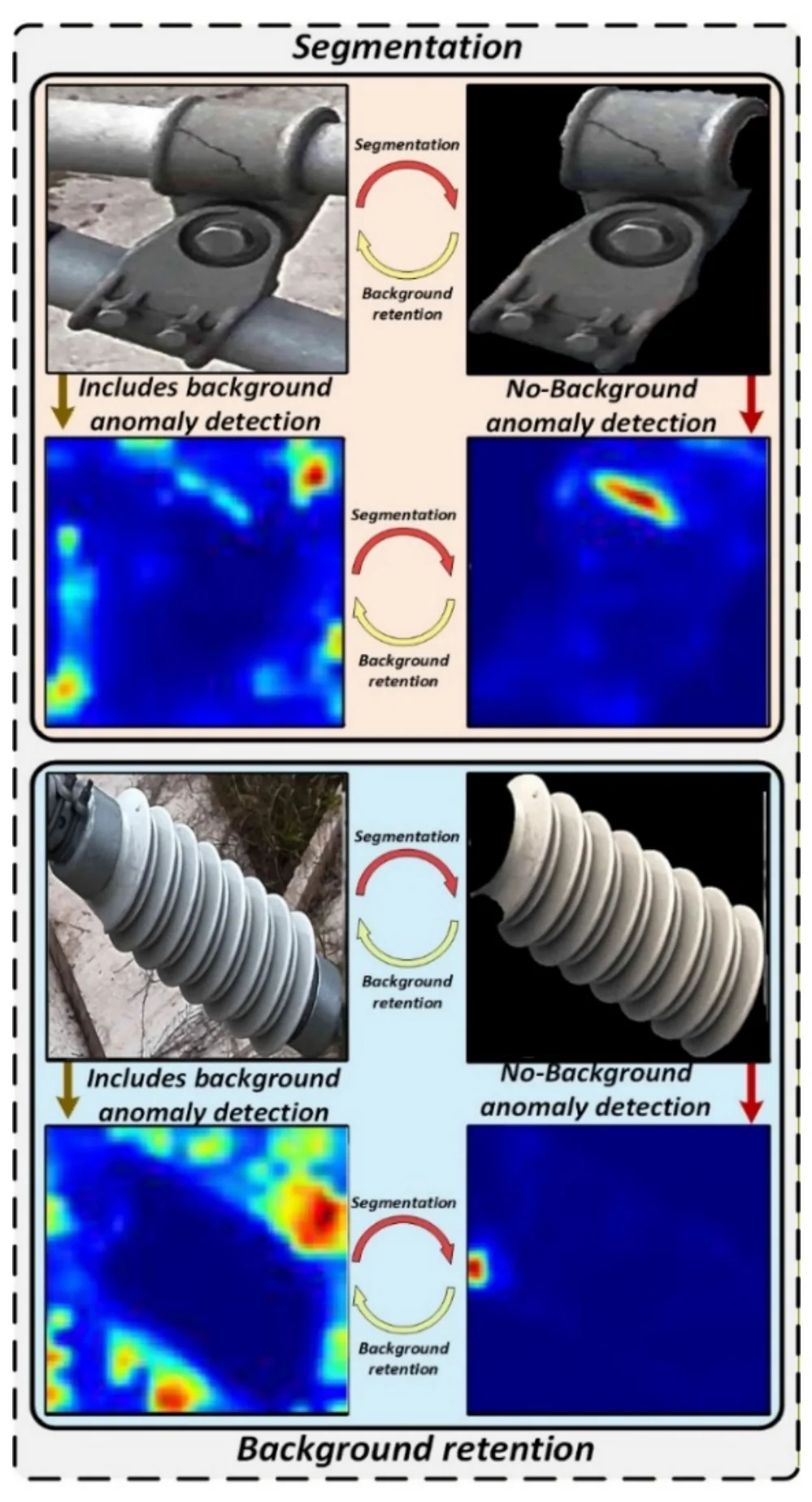
Comparison of the impact of foreground extraction strategies on anomaly localization. Columns correspond to Original Image, Anomaly Heatmap with Background, Anomaly Heatmap with Background Removed (MGAF).

To validate the necessity of the SAM-based structural isolation strategy within the MGAF framework, we conducted a visual comparative analysis between the “raw imagery with unsuppressed Environmental and Operational Variations (EOVs)” and the “EOV-suppressed structural ROIs via masking.” The diagnostic results are illustrated in Figure 7.

From the damage localization heatmaps in Figure 7, significant differences can be clearly observed:

(1) With Unsuppressed EOVs: The diagnostic heatmap exhibits severe “Diagnostic Distraction.” High-response regions are not focused on genuine structural damages within the components, but are widely distributed across complex background clutter, such as ballast, rail tracks, and vegetation. This occurs because the probabilistic model attempts to mathematically fit these high-frequency and irregular environmental variations, resulting in severe healthy baseline modeling deviations and, consequently, a massive influx of false alarms.
(2) EOV-Suppressed (SAM-Masked): In contrast, the inputs processed by SAM (i.e., the isolated structural ROIs fed into MGAF) yield heatmaps that accurately concentrate the analytical focus on actual structural degradation regions, such as insulator fatigue cracks or housing defects. Meanwhile, the masked environmental areas maintain a stable, interference-free low-response baseline.

This comparative analysis clearly demonstrates that the severe EOVs introduced by UAV aerial perspectives fatally interfere with the generative model’s ability to learn the healthy baseline structural distribution of the components. Therefore, integrating SAM-guided EOV suppression into the MGAF framework is an indispensable prerequisite for eliminating environmental interference and ensuring the monitoring system focuses exclusively on the critical load-bearing structural topologies and localized damage indicators.

#### 3.2.6. Ablation Analysis of Anomaly Detection Module

To verify the contribution of each core functional module within the proposed framework, comprehensive ablation studies were conducted on the CSCUD dataset, with results summarized in Table 6.

(1) Effectiveness of CSCSAM-based Structural Isolation: The first two rows in Table 6 compare the baseline model with the model equipped with the CSCSAM module. Without structural isolation, the baseline achieves an image-level AUROC of 85.5% and a pixel-level AP of only 14.5%. This indicates that complex background information and environmental interference in UAV-based catenary images significantly affect the modeling of normal structural patterns. After introducing CSCSAM, the image-level AUROC increases from 85.5% to 94.2%, while the pixel-level AP improves from 14.5% to 26.8%. The significant improvement demonstrates that structural ROI extraction effectively suppresses irrelevant environmental variations and provides more discriminative structural features for subsequent anomaly detection.
(2) Contribution of the Asymmetrical Parallel Flow Architecture: The third row evaluates the influence of the proposed ASYM module based on the CSCSAM-enhanced model. After introducing the asymmetrical flow architecture, the image-level AUROC increases from 94.2% to 95.6%, while the pixel-level AP improves from 26.8% to 29.4%. This improvement verifies that assigning different flow depths to multi-scale features contributes to more effective probability distribution modeling. Specifically, the asymmetrical design reduces unnecessary transformations for high-resolution features while maintaining sufficient representation capability for high-level semantic features, thereby improving anomaly detection performance and reducing the risk of overfitting to complex UAV image textures.
(3) Contribution of Cross-Scale Feature Fusion: The fourth row investigates the effect of CSFF by introducing the cross-scale feature interaction module into the CSCSAM-based framework. The results show that CSFF improves the image-level AUROC from 94.2% to 96.1% and significantly increases the pixel-level AP from 26.8% to 35.6%. The remarkable improvement in pixel-level localization indicates that effective interaction between different feature scales is essential for detecting fine-grained structural defects. By combining high-level semantic information with low-level texture details, CSFF enables the model to better distinguish actual defects from normal local appearance variations.
(4) Effectiveness of Morphological Edge Suppression: The fifth row evaluates the contribution of MES under the CSCSAM-based framework. After adding MES, the image-level AUROC increases to 97.1%, and the pixel-level F1-score improves from 46.2% to 48.4% compared with the CSFF-based configuration. This demonstrates that MES mainly contributes to localization refinement. By suppressing abnormal responses around structural boundaries and segmentation interfaces, MES reduces false-positive regions and improves the spatial consistency between predicted anomaly regions and actual defect areas.
(5) Effect of Combining Multiple Components: To further verify the complementary relationship among different modules, the sixth row removes the ASYM module while retaining CSCSAM, CSFF, and MES. This configuration achieves an image-level AUROC of 98.1% and a pixel-level AP of 38.2%, demonstrating that CSCSAM, CSFF, and MES together already provide strong anomaly detection capability. However, after incorporating the complete MGAF framework with all four components, the final model achieves the best performance, reaching an image-level AUROC of 98.9%, an image-level AP of 97.6%, and a pixel-level AP of 40.1%. Compared with the baseline model, the complete MGAF framework improves the image-level AUROC by 13.4 percentage points and the pixel-level AP by 25.6 percentage points. These results confirm that each component contributes complementary advantages: CSCSAM reduces environmental interference, ASYM improves multi-scale feature distribution modeling, CSFF enhances fine-grained defect localization, and MES suppresses boundary-related false responses. The combination of these modules enables MGAF to achieve robust and accurate unsupervised structural health monitoring under complex UAV inspection conditions.

#### 3.2.7. Analysis of the Model’s Generalization Performance

**Figure 8:**
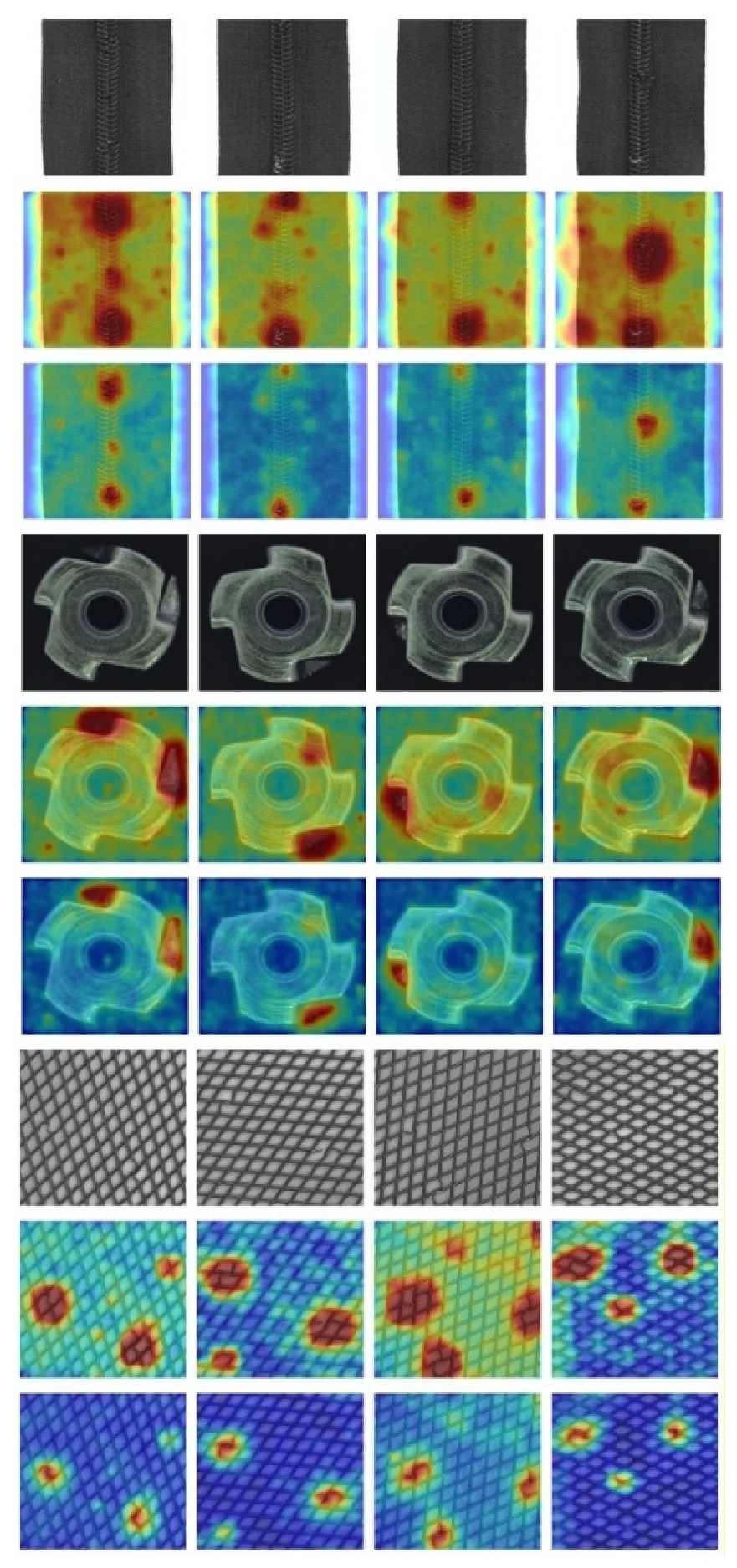
Generalization visualization on MVTec AD dataset. The proposed MGAF accurately localizes defects across diverse industrial categories, including texture interruptions in Grid, surface scratches on Metal Nut, and structural defects in Zipper.

To further evaluate the generalization capability of the MGAF framework across diverse industrial contexts, we conducted extended experiments on the MVTec AD benchmark, complementing the specialized CSCUD dataset. We selected three representative categories with distinct structural topologies: Grid (representing periodic cellular textures), Metal Nut (representing rigid precision fasteners), and Zipper (representing complex multi-component assemblies). These objects share fundamental geometric and textural similarities with railway catenary components (e.g., ceramic insulator sheds and metallic fasteners), providing a rigorous testbed for the model’s cross-domain robustness. The quantitative results are summarized in Table 7, and the corresponding visual results are presented in Figure 8.

The empirical data demonstrates that MGAF sustains SOTA-level performance across these diverse structural forms:

Grid (Periodic Cellular Structures): MGAF exhibited superior texture-modeling fidelity. Leveraging the asymmetrical flow architecture, the model effectively captures the periodic high-frequency features of the lattice. This makes the framework highly sensitive to structural discontinuities that disrupt textural homogeneity, such as metallic contamination or grid fractures, proving its applicability for general surface integrity inspections.

Metal Nut (Rigid Precision Fasteners): For metallic fasteners, defects typically manifest as subtle morphological deviations (e.g., notches or surface abrasions). Through the Cross-Scale Feature Fusion (CSFF) strategy, MGAF synergizes macroscopic geometric contours with microscopic surface details. This enables precise pixel-level localization of infinitesimal structural anomalies that are often overlooked by single-scale models.

Zipper (Complex Multi-component Assemblies): The Zipper category involves intricate backgrounds and non-rigid structural deformations. MGAF proved robust in isolating the critical structural features from complex interference, demonstrating its exceptional resistance to Environmental and Operational Variations (EOVs) without overfitting to domain-specific artifacts.

In summary, MGAF is not merely a bespoke solution for railway infrastructure; its core architectural principles—specifically the asymmetrical probabilistic flow and multi-scale evidence integration—possess the versatility to address a broad spectrum of industrial structural health monitoring challenges, spanning periodic textures, rigid bodies, and complex non-rigid assemblies. In summary, MGAF is not only a solution tailored for railway inspection, but its core architecture (asymmetrical flow and multi-scale fusion) has the potential to handle various industrial forms (textures, rigid and non-rigid bodies), proving its versatility.

## 4. Discussion

The experimental results demonstrate that the proposed Mask-Guided Asymmetrical Flow (MGAF) framework provides an effective solution for unsupervised structural health monitoring of railway catenary components under complex field inspection conditions. Unlike conventional industrial anomaly detection scenarios, UAV-based railway inspection is inevitably affected by severe Environmental and Operational Variations (EOVs), including background clutter, illumination changes, and complex shooting perspectives. The superior performance of MGAF indicates that explicitly separating structural information from environmental interference is essential for reliable condition assessment in real-world infrastructure monitoring.

A key finding of this study is that the integration of foundation-model-based structural isolation with probabilistic anomaly modeling can significantly improve the robustness of unsupervised damage detection. The SAM-guided Region of Interest (ROI) extraction strategy provides a clean structural representation by suppressing irrelevant background information before feature modeling. This design reduces the probability that environmental patterns are incorrectly incorporated into the healthy structural distribution, thereby alleviating false alarms caused by complex outdoor scenes. Compared with conventional flow-based approaches that directly model complete images, MGAF establishes a more physically meaningful healthy baseline by constraining the learning process to structural components.

Another important observation is that the multi-scale asymmetric architecture plays a critical role in balancing detection accuracy and computational efficiency. Railway catenary degradation exhibits strong scale diversity, ranging from macroscopic structural deformation to microscopic defects such as fatigue cracks and fastener looseness. The proposed asymmetrical parallel flow mechanism adaptively allocates different modeling capacities to different feature scales, avoiding excessive transformation of high-resolution features while maintaining sufficient representation ability for semantic structural information. Furthermore, the Cross-Scale Feature Fusion (CSFF) mechanism enables local damage indicators to be interpreted within global structural contexts, which effectively improves localization accuracy and reduces misclassification caused by isolated texture variations.

From an engineering perspective, MGAF demonstrates considerable potential for practical deployment in intelligent railway maintenance systems. The achieved image-level detection performance, pixel-level localization capability, and real-time inference speed indicate that the proposed framework can support automated inspection and early warning applications. In particular, the ability to identify small-scale degradation without requiring large quantities of defective samples is highly valuable for railway infrastructure, where abnormal events are rare but potentially safety-critical. Therefore, MGAF provides a feasible pathway toward condition-based maintenance by reducing dependence on manual inspection and improving the efficiency of large-scale infrastructure monitoring.

Despite these advantages, several limitations remain. First, although the CSCUD dataset contains diverse real-world UAV inspection scenarios, further validation using larger-scale datasets collected from different railway environments, weather conditions, and operational periods is still required to evaluate long-term robustness. Second, the current framework mainly focuses on spatial appearance information from individual inspection images and does not explicitly model temporal degradation trends. In practical railway maintenance, damage evolution over time is an important indicator for predictive decision-making. Future research will therefore investigate the integration of sequential UAV inspection data, temporal representation learning, and degradation prediction mechanisms to extend MGAF from anomaly identification toward proactive maintenance planning.

## 5. Conclusions

This paper proposed a Mask-Guided Asymmetrical Flow (MGAF) framework for unsupervised structural health monitoring of railway catenary components using UAV inspection imagery. By combining SAM-guided structural ROI extraction with an asymmetrical Normalizing Flow architecture, MGAF effectively suppresses complex Environmental and Operational Variations while establishing a reliable healthy structural representation. Furthermore, the proposed Cross-Scale Feature Fusion mechanism enhances the interaction between global structural semantics and local damage characteristics, enabling accurate identification and localization of multi-scale structural anomalies.

Inspired by the biological visual perception system, MGAF incorporates several biomimetic principles into the design of an intelligent inspection framework. Similar to the selective attention mechanism of the human visual system, the CSCSAM module focuses diagnostic processing on relevant structural regions while suppressing environmental interference. Furthermore, the asymmetrical flow architecture follows the hierarchical processing characteristics of biological vision, where different levels of visual information are processed through pathways with different computational capacities. The CSFF module mimics the multi-scale integration mechanism of biological perception by combining local details and global structural information, while MES is inspired by biological edge perception for enhancing structural boundary awareness. These biomimetic principles enable MGAF to achieve efficient and robust anomaly detection under complex UAV inspection conditions.

Extensive experiments conducted on the field-acquired CSCUD dataset and the public MVTec benchmark demonstrate that MGAF achieves superior performance in both anomaly detection and pixel-level damage localization. The framework achieves an image-level AUROC of 98.9%, a pixel-level AP of 40.1%, and maintains efficient inference capability suitable for real-time inspection applications. These results verify the effectiveness of the proposed approach in addressing the challenges of small-sample learning, complex environmental interference, and multi-scale damage characterization in railway infrastructure monitoring.

Overall, MGAF provides a robust and scalable vision-based non-destructive evaluation framework for intelligent railway maintenance. By combining foundation models, probabilistic generative learning, and multi-scale structural reasoning, the proposed method offers a promising direction for future autonomous inspection systems. Further studies will focus on incorporating temporal inspection sequences and predictive degradation modeling to achieve more comprehensive and intelligent railway infrastructure health management.

## Figures and Tables

**Table 1:** Distribution of positive and negative samples for catenary components.

Categories	Number of Positive and Negative Samples	
	Normal Samples (Training)/(Testing)	Anomalous Samples (Testing)
Casing base (C1)	1125/125	318
Connector sleeve (C2)	765/85	206
Insulator (C3)	1053/117	316
Locator base (C4)	1152/128	294
Locator tube connector (C5)	1332/148	342
screw_clamp (C6)	2990/136	238
Sleeve double ear (C7)	2160/240	558

**Table 2:** The results of per-class anomaly detection for catenary components.

Methods Categories	Indicators: (Img-AUROC/Img-AP/Img-F1)							
	UniAD [25]	RD4AD [30]	DiAD [31]	SimpleNet [32]	DeSTSeg [33]	MambaAD [26]	Dinomaly [34]	MGAF (Ours)
C1	99.3/99/98.9	98.6/98.9/98.4	98.7/95.5/96.3	100/100/99.1	97.7/98.6/97.9	99.1/99.9/98.8	99/99/98.9	100/100/99.9
C2	98.7/99/98.4	99.1/99/99	98.8/97.8/98.5	98.8/97.2/98.6	98.2/97.9/98	99.3/99/99	99.4/99/98.8	100/100/99.8
C3	98.4/98/98.1	97.5/98.2/97.9	96.5/98.6/95.3	98.8/97.7/97.9	97.9/97.8/97	100/98.7/98.9	98.7/99.5/98.8	100/100/99.9
C4	94.8/98.7/97.3	98.4/97.4/97.3	94.1/96.8/96.8	95.0/98.3/96.2	87.5/93.8/89.9	98.3/99.2/97.9	96.6/97.9/98.1	98.9/96.9/97.7
C5	98.4/98.9/97.8	100/100/98.8	98.4/99.2/97.5	98.2/95/96.4	92.9/97.4/94.5	98.9/99/99	99.2/100/99	98.8/98.3/98.7
C6	96.7/94.1/94	93.9/92.9/93.2	97.1/91.3/95.2	96.9/96.5/95.9	86.7/90.1/91.5	98.1/94.7/96.5	98.3/94.4/95.5	98.2/96.6/95.9
C7	94.6/89.2/92.5	84.1/83.8/83.8	94.3/97.4/93	96.8/92.4/94.2	86.7/78.8/75.7	97.6/95.4/94.1	100/94.8/95.3	97.1/94.7/96.4
Mean	97.2/96.4/96.4	95.7/95.4/95.2	96.9/96/96	97.7/96.7/96.8	91.8/93.1/92	98.7/97.3/97.6	98.7/97.4/97.5	98.9/97.6/98.12

**Table 3:** The results of per-class anomaly localization for catenary components.

Methods Categories	Indicators: (Pixel-AUROC/Pixel-AP/Pixel-F1)							
	UniAD [25]	RD4AD [30]	DiAD [31]	SimpleNet [32]	DeSTSeg [33]	MambaAD [26]	Dinomaly [34]	MGAF (Ours)
C1	96.2/32.3/54.2	95.9/33.4/54.4	96.5/25.5/56.4.	95.3/26.3/53.7	91.5/30.2/51.2	96.9/39/56.3	98.3/43.4/57.2	98.2/44.5/59.9
C2	96.2/26.7/56.1	96.7/30.8/56	96.2/45.5/54.7	96.1/23.1/54.6	97.9/58/56.5	96.8/40.8/56.8	96.8/42.4/55.2	98.9/43.4/57.8
C3	92.7/34.2/52	96.2/39.4/54.7	93.3/53.4/52.7	96.7/49.2/56.6	93.9/57.7/53.2	94.7/36.7/53	99.8/61.2/58.6	99.5/65.5/59.9
C4	95.3/14.3/54.2	97/19.3/55.5	94.7/21.7/53.2	96.4/19.1/55	95.8/23.1/55.6	96.9/21.6/55.1	97.9/26.5/57	98.3/26.3/59.9
C5	62.5/7.8/31.2	95.1/20.2/54.5	97.6/10.9/57.5	99.3/29.6/59.1	84.3/15.3/44.2	97/27.2/56	97.2/28.7/56.3	98.4/30.6/58.8
C6	61.1/8.6/29.8	93.9/12.7/53.5	94.4/35.5/53.1	94.2/11.3/52.3	94.4/23.3/53.2	96.6/26.2/54.8	97.8/30.6/56.3	97.6/33.9/58.6
C7	94.9/14.5/53.5	82.9/9.6/38.6	94.4/18.2/51.6	94.3/15.4/51.7	87/13.6/42.6	93.4/15.3/51.5	96.2/26.1/53.7	96.9/39/56.9
Mean	85.4/20.5/50.5	91.5/23/50	93.6/27.4/52.6	94.4/24.9/53	88.5/29.7/47.5	94.5/29.6/53.1	95.5/35.6/54.3	96.3/40.1/56.7

Note: The values in Table 2 and Table 3 represent the quantitative results of anomaly detection and localization, respectively.

**Table 4:** The results of multi-class anomaly detection for catenary components.

Indicators Method	Image-Level			Pixel-Level		
	I-AUROC	I-AP	I-F1	P-AUROC	P-AP	P-F1
MambaAD [26]	98.7 ± 0.1	97.3 ± 0.2	97.6 ± 0.2	94.5 ± 0.3	29.6 ± 0.5	53.1 ± 0.4
Dinomaly [34]	98.7 ± 0.2	97.4 ± 0.2	97.5 ± 0.1	95.5 ± 0.2	35.6 ± 0.6	54.3 ± 0.5
DeSTSeg [33]	91.8 ± 0.4	93.1 ± 0.3	92 ± 0.4	88.5 ± 0.5	29.7 ± 0.7	49.5 ± 0.6
SimpleNet [32]	97.7 ± 0.2	96.7 ± 0.2	96.8 ± 0.3	94.4 ± 0.3	24.9 ± 0.8	53 ± 0.5
DiAD [31]	96.9 ± 0.3	96.0 ± 0.2	96 ± 0.4	93.6 ± 0.4	27.4 ± 0.7	52.6 ± 0.6
UniAD [25]	97.2 ± 0.2	96.4 ± 0.3	96.4 ± 0.2	85.4 ± 0.6	20.5 ± 0.9	47.5 ± 0.8
RD4AD [9]	95.7 ± 0.3	95.4 ± 0.4	95.2 ± 0.3	91.5 ± 0.4	23.0 ± 0.7	50.0 ± 0.7
SDMAE [35]	96.3 ± 0.3	95.3 ± 0.3	94.3 ± 0.5	93.1 ± 0.4	26.9 ± 0.8	51.8 ± 0.6
Diff-AD [36]	97.4 ± 0.2	96.7 ± 0.2	96.2 ± 0.4	87.4 ± 0.5	27.2 ± 0.9	50.3 ± 0.7
MGAF(ours)	98.9 ± 0.1	97.6 ± 0.2	98.1 ± 0.1	96.3 ± 0.1	40.1 ± 0.2	56.7 ± 0.3

Note: All results are reported as mean ± standard deviation over ten independent runs with different random seeds.

**Table 5:** Comparison experiment of computational efficiency between sota methods.

Indicators Method	mAD	FLOPs (G)	Params (M)	Times (s)
MambaAD [26]	78.5	8.5	26.1	0.052
Dinomaly [34]	79.8	104.9	133.2	0.155
DeSTSeg [33]	74.1	123.2	35.6	0.173
SimpleNet [32]	77.3	16.3	73.3	0.061
DiAD [31]	77.1	45.1	133.1	1.125
UniAD [25]	73.9	3.8	242.7	0.023
RD4AD [9]	75.1	39.4	151.7	0.098
SDMAE [35]	76.3	76.4	136.2	0.134
Diff-AD [36]	75.9	80.3	125.6	0.156
MGAF(ours)	81.3	65.9	216.50	0.07

Note: mAD represents the average value of the six indicators.

**Table 6:** The results of ablation experiments.

Modle				Image-Level	Pixel-Level
MES	CSFF	ASYM	CSCSAM	I-AUROC/I-AP/I-F1	P-AUROC/P-AP/P-F1
-	-	-	-	85.5/81.2/84.5	88.2/14.5/18.3
-	-	-	✓	94.2/91.5/93.8	94.1/26.8/34.2
-	-	✓	✓	95.6/93.1/95.2	94.5/29.4/37.5
-	✓	-	✓	96.1/94.2/95.0	95.2/35.6/46.2
✓	-	✓	✓	97.1/95.3/96.2	95.7/35.8/48.4
✓	✓	-	✓	98.1/96.2/97.0	96.0/38.2/53.0
-	✓	✓	✓	98.2/96.5/97.5	95.8/34.1/48.6
✓	✓	✓	✓	98.9/97.6/98.1	96.3/40.1/56.7

Note: The abbreviations in the table are defined as follows: CSCSAM denotes the SAM-based Catenary System Component Preprocessing (Background Removal); ASYM represents the Asymmetrical Parallel Flow Architecture; CSFF stands for the Cross-Scale Feature Fusion module; and MES refers to the Morphological Edge Suppression strategy.

**Table 7:** The results of multi-class anomaly detection on different datasets.

Indicators Method	Image-Level			Pixel-Level		
	I-AUROC	I-AP	I-F1	P-AUROC	P-AP	P-F1
Grid	99.5	99.8	99.1	99.3	51.2	54.1
Metal Nut	99.9	99.9	99.9	99.2	64.4	68.1
Zipper	99.9	100	99.2	99.1	63.4	69.4
Mean	99.8	99.9	99.4	99.2	59.7	63.9

## Data Availability

The original contributions presented in this study are included in the article. The dataset generated during this study is not publicly available due to data management considerations. Further inquiries can be directed to the corresponding author.

## Abbreviations

The following abbreviations are used in this manuscript:

SHM	Structural Health Monitoring
UAV	Unmanned Aerial Vehicle
EOVs	Environmental and Operational Variations
NDE	Non-Destructive Evaluation
MGAF	Mask-Guided Asymmetrical Flow
SAM	Segment Anything Model
ROI	Region of Interest
UDD	Unsupervised Damage Detection
NLL	Negative Log-Likelihood
NF	Normalizing Flow
CSFF	Cross-Scale Feature Fusion
MES	Morphological Edge Suppression
WRN	Wide Residual Network
CNN	Convolutional Neural Network
AP	Average Precision
AUROC	Area Under the Receiver Operating Characteristic Curve
F1-score	F1 Score
FLOPs	Floating Point Operations
CSCUD	Catenary Support Component UAV Dataset
CBM	Condition-Based Maintenance
MVTec AD	MVTec Anomaly Detection Dataset
SOTA	State-of-the-Art

## References

[1] Yang H., Liu Z., Liu W., Wang H., Zhang Y., Wang H. (2026). Graph-MDETR: A graph-guided Mamba-DETR network for UAV catenary support components detection in electrified railways. IEEE Trans. Intell. Transp. Syst..

[2] Wang H., Song Y., Yang H., Liu Z. (2025). Generalized Koopman neural operator for data-driven modelling of electric railway pantograph-catenary systems. IEEE Trans. Transp. Electrif..

[3] Ma N., Yang H., Liu Z., Pi Y., Cui H., Wang H., Song Y. (2026). Hybrid Spiking-Visual-Language Framework for Unmanned Aerial Vehicle-Based Inspection and Anomaly Detection of Catenary Support Components in Electrified Railways. Eng. Appl. Artif. Intell..

[4] Zhang G., Liu Z. (2014). Fault detection of catenary insulator damage/foreign material based on corner matching and spectral clustering. Chin. J. Sci. Instrum..

[5] Yang H.-M., Liu Z.-G., Han Z.-W., Han Y. (2013). Foreign body detection between insulator pieces in electrified railway based on affine moment invariant. J. China Railw. Soc..

[6] Karakose E., Gencoglu M.T., Karakose M., Yaman O., Aydin I., Akin E. (2018). A new arc detection method based on fuzzy logic using S-transform for pantograph–catenary systems. J. Intell. Manuf..

[7] Zhong J., Liu Z., Yang C., Wang H., Gao S., Núñez A. (2020). Adversarial reconstruction based on tighter oriented localization for catenary insulator defect detection in high-speed railways. IEEE Trans. Intell. Transp. Syst..

[8] Zhang Y., Zhong J., Liu Z., Han Z. (2023). ECF-STPM: A robust crack detection method for railway catenary components. IEEE Trans. Instrum. Meas..

[9] Chen J., Liu Z., Wang H., Núñez A., Han Z. (2017). Automatic defect detection of fasteners on the catenary support device using deep convolutional neural network. IEEE Trans. Instrum. Meas..

[10] Xie W., Yang H., Shi L., Liu Z. (2024). MTA-Net: A one-stage detector based on a multiscale task-aligned network for catenary support components. IEEE Trans. Instrum. Meas..

[11] He Z., Tan G., Zhou E., Li E., Hu B., Li C., Li B. (2026). Enhancing fault diagnosis with a hybrid attention mechanism and spatio-temporal feature mining model using small sample data. Struct. Health Monit..

[12] Yang G., Jiang Y., Wang S., Chen K. (2026). VinsFusion-Line: Binocular vision inertial navigation real-time SLAM system based on line features. Cybern. Syst..

[13] Zhang Q., Liu L., Liu T., Ma L., Wang Y. (2026). WaveletMixer: When wavelet transform meets self-attention in single image super-resolution. IEEE Trans. Circuits Syst. Video Technol..

[14] Chen Z., Li N., Zhang H., Xu S., Chen W., Zhang J., Fu L., Hu H., Peng Y., Deng F. (2025). Direction-aware attention for detail-preserving terahertz image super-resolution. Nondestruct. Test. Eval..

[15] Zhuang K., Deng B., Chen H., Jiang L., Li Y., Hu J., Lam H. (2026). An enhanced deep intelligent model with feature fusion and ensemble learning for the fault diagnosis of rotating machinery. Struct. Health Monit..

[16] Xiang W., Liu S., Li H., Yang C., Cao S., Zhang K. (2026). Prototype-assisted multiscale graph representation learning-based mechanical fault detection method under complex operating conditions. Struct. Health Monit..

[17] Li Y., Zhao Z., Sun L., Guo T., Yang B. (2026). A geometrical morphology-enhanced computer vision approach for structural health assessment. Struct. Health Monit..

[18] Sreekala K., Maniraj S., Singh A., Singh A.P., Pyingkodi M., Inthiyaz S. (2026). Enhancing medical diagnosis through multimodal image fusion: A novel approach using modified swin-based cross attention fusion. Cybern. Syst..

[19] Reddy K M.S., Nelakuditi U.R. (2025). Illustration of image registration-based novel segmentation and classification model using multimodal medical images with 3d-trrsegnet and adaptive ran. Cybern. Syst..

[20] Deng F., Jin J., Chen X., An Z., Du Y. (2026). Enhancing automated health monitoring of road infrastructure through a hierarchical robust deep learning approach. Struct. Health Monit..

[21] U.H. D., Kumar J.P. (2025). Enhanced transfer learning-based CNN for abnormal human activity detection in video surveillance using spatial-temporal features. Cybern. Syst..

[22] Shajeena J., Govindasamy B., Gnanasundaram M., Robinson Joel M. (2026). Mobile-Le Harmonic Fusion Network for Object Recognition and SiamMoT Based Multi-Object Tracking Using Video Surveillance. Cybern. Syst..

[23] Chen Z., Zhang H., Fan M., Li N. (2026). Wavelet-Guided Dynamic Transformer for High-Fidelity Super-Resolution in Terahertz Imaging Systems. IEEE Sens. J..

[24] Lyu S., Mo D., Wong W.K. (2024). REB: Reducing biases in representation for industrial anomaly detection. Knowl.-Based Syst..

[25] You Z., Cui L., Shen Y., Yang K., Lu X., Zheng Y., Le X. (2022). A unified model for multi-class anomaly detection. Adv. Neural Inf. Process. Syst..

[26] He H., Bai Y., Zhang J., He Q., Chen H., Gan Z., Wang C., Li X., Tian G., Xie L. (2024). Mambaad: Exploring state space models for multi-class unsupervised anomaly detection. Adv. Neural Inf. Process. Syst..

[27] Yan J., Chen B., Zhang F., Cheng Y., Wang H., Wang H., Wang M., Li T., Zhang W. (2026). Meta-Learning-Based Graph Convolutional Wavelet Network for Intelligent Dynamic Modeling of High-Speed Rail Subsystems. IEEE Trans. Veh. Technol..

[28] Ma N., Yang H., Liu Z., Cui H., Wang H., Shi L. (2026). Support structure detection of railway catenary systems on UAVs using spike-based brain-inspired neural network. IEEE Trans. Instrum. Meas..

[29] Luo M., Zhang T., Wei S., Ji S. (2024). SAM-RSIS: Progressively adapting SAM with box prompting to remote sensing image instance segmentation. IEEE Trans. Geosci. Remote Sens..

[30] Deng H., Li X. (2022). Anomaly detection via reverse distillation from one-class embedding. Proceedings of the 2022 IEEE/CVF Conference on Computer Vision and Pattern Recognition (CVPR), New Orleans, LA, USA, 18–24 June 2022.

[31] He H., Zhang J., Chen H., Chen X., Li Z., Chen X., Wang Y., Wang C., Xie L. (2024). A diffusion-based framework for multi-class anomaly detection. Proc. AAAI Conf. Artif. Intell..

[32] Liu Z., Zhou Y., Xu Y., Wang Z. (2023). Simplenet: A simple network for image anomaly detection and localization. Proceedings of the 2023 IEEE/CVF Conference on Computer Vision and Pattern Recognition (CVPR), Vancouver, BC, Canada, 17–24 June 2023.

[33] Zhang X., Li S., Li X., Huang P., Shan J., Chen T. (2023). Destseg: Segmentation guided denoising student-teacher for anomaly detection. Proceedings of the 2023 IEEE/CVF Conference on Computer Vision and Pattern Recognition (CVPR), Vancouver, BC, Canada, 17–24 June 2023.

[34] Guo J., Lu S., Zhang W., Chen F., Li H., Liao H. (2025). Dinomaly: The less is more philosophy in multi-class unsupervised anomaly detection. Proceedings of the 2025 IEEE/CVF Conference on Computer Vision and Pattern Recognition (CVPR), Nashville, TN, USA, 10–17 June 2025.

[35] Ye S., Yu J. (2025). SDMAE: A self-supervised learning method based on a self-distillation masked autoencoder for anomalous sound detection. Appl. Acoust..

[36] Zhang H., Wang Z., Zeng D., Wu Z., Jiang Y.-G. (2025). DiffusionAD: Norm-guided one-step denoising diffusion for anomaly detection. IEEE Trans. Pattern Anal. Mach. Intell..

